# Towards a multidimensional measure of well-being: cross-cultural support through the Italian validation of the well-being profile

**DOI:** 10.1186/s40359-023-01485-9

**Published:** 2023-12-13

**Authors:** L. Francesca Scalas, Ernesto Lodi, Paola Magnano, Herbert W. Marsh

**Affiliations:** 1 Dipartimento di Pedagogia, Psicologia, Filosofia, Università degli Studi di Cagliari, Via Is Mirrionis, 1- Cagliari (Loc. Sa Duchessa), Cagliari, 09123 Italy; 2 Università degli Studi di Sassari, Sassari, Italy; 3 Università degli Studi di Enna “Kore”, Enna, Italy; 4 Australian Catholic University, Sydney, Australia

**Keywords:** Multidimensional well-being scale, Well-being profile, Italian validation, Factor analysis, Bifactor analysis

## Abstract

**Background:**

The Well-being Profile (WB-Pro) is a multi-item and multidimensional instrument with strong psychometric properties and a solid theoretical grounding. It includes aspects of hedonic and eudaimonic well-being that can be used at the individual and social levels.

**Method:**

We developed the Italian version through back-translation procedures. The aim of this study is to validate the WB-Pro in Italian as well as to better understand its multidimensionality through bifactor analysis. A sample of 1451 participants (910 = women, 62.7%; age range: 18–70, M-age = 32.34, SD-age = 13.64) was involved.

**Results:**

The 15-factor structure was confirmed with CFA and ESEM and was invariant across gender, age, and education. We examined convergent and discriminant validity and a bifactorial representation. Short versions of the WB-Pro were tested.

**Discussion:**

Even though a few items of the Italian version of the WB-Pro might benefit from revision (e.g., *clear-thinking* scale), this study confirms the theoretical and empirical strength of the WB-Pro.

**Conclusions:**

This study supports the WB-Pro validity and usefulness in studying well-being and for professional psychological applications to assess well-being in both individuals and groups.

**Supplementary Information:**

The online version contains supplementary material available at 10.1186/s40359-023-01485-9.

## Background

In this study we present an Italian validation of the Well-being Profile (WB-Pro); moreover, we use bifactor analysis to better understand its multidimensionality.

Marsh and colleagues [1], based on the perspective of well-being as positive mental health, developed the WB-Pro that includes both hedonic and eudaimonic aspects of well-being. The 15 dimensions of the instrument (i.e., autonomy, clear thinking, competence, emotional stability, empathy, engagement, meaning, optimism, positive emotion, positive relationships, prosocial behavior, resilience, self-acceptance, self-esteem, and vitality; see supplemental materials for a description) include aspects of emotional well-being, psychological well-being, and social well-being [2]. The instrument had excellent psychometric properties in the U.S. samples used to develop the scale. However, currently, there are no other validation studies. The associations with various demographic (e.g., level of education, marital status) and psychological (e.g., traits of personality, psychological need satisfaction, and frustration) criteria support the convergent and discriminant validity of the WB-Pro. Moreover, using a machine-learning algorithm, Marsh and colleagues [1] developed two short formative versions of the WB-Pro that can be used in case of limitations on survey length. Hence the interest in validating the WB-Pro in cultural contexts other than English-speaking countries contributes to the cross-cultural evidence of this multidimensional scale that is useful to researchers, practitioners, and policymakers.

## Well-being: a complex and multifaced construct

In the 21st century, the interest in well-being has grown exponentially in scientific studies and professional psychological practice. The orientation toward what makes a life happy and valuable, and toward the promotion of subjective and psychological well-being, provides a balance to a focus on what was wrong in people and what caused discomfort. What emerges from these studies (e.g., [2–4]) is that well-being is a complex and multidimensional construct, concerning the emotional, cognitive, behavioral, personal, and social spheres of human experience and their optimal functioning. Moreover, public policies have been paying growing attention to evaluating subjective well-being, focusing on additional elements beyond the conditions and objective indices of well-being [3]. Nonetheless, many nations consider well-being only through the dimension of life satisfaction or satisfaction with relationships, neglecting the multidimensionality highlighted in the recent literature on well-being [3]. As well as the measurement of mental illness cannot be reduced to a single dimension - such as depression or anxiety -, similarly, subjective well-being is complex and requires measures beyond life satisfaction and happiness [3]. Therefore, a multidimensional measure of well-being is useful to assess the effectiveness of public policies aimed at health and supporting investments in this direction.

## The hedonic and eudaimonic approach to well-being

There are two traditions in the study of well-being: the hedonic and eudaimonic approaches. These are sometimes seen as opposed, and other times as interrelated or complementary [5, 6]. The hedonic perspective is more related to pleasure and happiness, a balance between positive and negative emotions. According to Diener, subjective well-being (SWB)^1^ has two components: a cognitive one represented by life satisfaction, and an affective one defined by the presence of positive affect and the absence of negative affect [7]. Thus, SWB can be considered an expression of the hedonic perspective. “SWB reflects an overall evaluation of the quality of a person’s life from her or his own perspective…and refers to the extent to which a person believes or feels that his or her life is going well” [8, p. 1].

On the other hand, the eudaimonic perspective concerns perceptions of exploiting one’s abilities and potential. Within the eudaimonic view, Ryff’s model of psychological well-being (PWB) shifts to what people think or put into action by pursuing the six ideals of life (autonomy, self-acceptance, personal growth, purposes of life, environmental control, positive relationships) [9]. Waterman and colleagues (e.g. [10, 11]) labelled the subjective experiences of eudaimonia as feelings of personal expressiveness. They found that these feelings are present when people follow their true selves, act to enhance their best potential, and pursue their purpose in life.

Self-determination theory [12] is also an eudaimonic conceptualization of well-being. It postulates that well-being results from satisfying three basic psychological needs (autonomy, competence, and relatedness). The satisfaction or frustration of basic needs predicts the levels of individuals’ well-being (in case of satisfaction) and psychological distress (in case of frustration) [13, 14].

## Towards a holistic model of well-being

The need to overcome opposing visions of the subjective experience of well-being has led researchers to seek a combination of hedonic and eudaimonic well-being [4, 15]. An attempt to combine hedonic and eudaimonic perspectives can be traced to Lent and Brown’s [16] socio-cognitive model of well-being. They sought to overcome treating SWB as a “context-free” construct, applying SWB to domain-specific valuable contexts, such as the work or training context. Su, Tay & Diener [17] presented a model of 18 aspects of positive functioning. These included dimensions of positive well-being, SWB, and other aspects such as relationships, meaning, engagement, mastery, optimism, and autonomy. Even though this compilation of well-being components was useful from a pragmatic perspective, it lacked a unifying model that was theoretically grounded [1].

Since 1948 World Health Organization defines positive mental health as a state of well-being in which individuals realize their abilities, can manage normal stressful life situations, and can work productively as active members of their community [18]. Thus, the idea of well-being as positive mental health has a long history. For example, Jahoda [19] developed criteria for positive psychological functioning by contrasting psychological well-being characteristics with psychological ill-being characteristics.

Moving from the idea that mental health is more than simply the absence of mental illness, Keyes [2] proposed the operationalization of mental health as a syndrome of symptoms of positive feelings and positive functioning. Specifically, mental health is composed of three fundamental dimensions: “feelings of happiness and satisfaction with life (emotional well-being), positive individual functioning in terms of self-realization (psychological well-being), and positive societal functioning in terms of being of social value (social well-being)” [20, p. 110]. Therefore, the presence of mental health is described as flourishing, characterized by high levels of emotional, psychological, and social well-being. In contrast, the absence of mental health is described as languishing, characterized by low emotional, psychological, and social well-being levels. Moderate mental health represents individuals who neither flourish nor languish in life [2].

According to Huppert and So [21], well-being goes beyond a neutral point reflecting the absence of mental ill-being symptoms. Therefore, they proposed a classification of well-being composed of positive psychological characteristics that were the opposite of symptoms of common mental disorders such as depression and anxiety (e.g., happiness and hopefulness in contrast with specific depression symptoms, calmness in contrast with generalized anxiety) and identified ten aspects of positive feeling and functioning, integrating hedonic and eudaimonic well-being components.

## Measuring well-being

There is an increasing interest in measuring well-being from individual and collective perspectives. Thus, measuring well-being helps to monitor a nation’s “happiness” and the efficacy of policy decisions taken to improve the well-being of a nation. It is important to consider how well-being is conceptualized as this drives the construction of measuring instruments. The most widely used well-being measures can be classified as having a primarily hedonic or eudaimonic focus (see a detailed review of instruments in the supplemental materials).

In summary, instruments measuring only hedonic well-being or only eudaimonic well-being provide limited information on the nature and complexity of people’s well-being. Furthermore, even though the traditional distinction between the hedonic and the eudaimonic perspectives of well-being could be overcome by using a multidimensional instrument that comprises both points of view, such as the WEWMBS [22], measuring well-being through a unique total score does not provide sufficient information about the various dimensions that represent strengths or weaknesses in a person’s profile, thus limiting its use, especially in clinical settings.

### The well-being profile

Marsh and colleagues [1] developed a multidimensional multi-item measure of well-being. The instrument is based mainly on the dimensions (competence, emotional stability, engagement, meaning, optimism, positive emotion, positive relationships, resilience, self-esteem, and vitality) identified by Huppert and So [21]. However, it extends Huppert and So by adding new dimensions (empathy, prosocial behavior, self-acceptance, clear thinking, and autonomy) and developing multiple items for each construct, thus improving reliability. Some of these dimensions are related to sociability, consistent with other well-being perspectives in the social and community fields. For example, Keyes [2, 23] stressed that social acceptance, social integration, social contribution, social actualization, and social coherence are fundamental for well-being. Some studies also concerned the relationship between a sense of community (local, organizational, scholastic) and life satisfaction (e.g. [24]), work well-being [25], and school well-being (e.g. [26]). Therefore, some recent studies confirm that individual well-being is linked to the satisfaction of social needs contributing to good functioning and conditions of psychological prosperity for the community [27]. Therefore, the 15 dimensions of the WB-Pro are also in line with Keyes’ [2] model, including emotional well-being and positive psychological functioning (e.g. [9]), but also the social and interpersonal dimensions of well-being.

The 15-factor structure of the WB-Pro was confirmed with CFA (Confirmatory Factor Analysis) and ESEM (Exploratory Structural Equation Modeling) models and was invariant over time, gender, education, and age [1]. The scales showed good reliability (range: 0.81–0.93) and test–retest correlation (range: 0.73–0.85). Associations with relevant demographic variables (gender, age, education, marital status) were examined, as well as links with various psychological correlates to test discriminant (personality traits) and convergent (e.g., psychological needs of satisfaction and frustration, flourishing) validity [1]. Marsh and colleagues [1] also showed the ability of the WB-Pro to absorb the Flourishing’s and the WEMWBS’ items, thus calling into question the claimed unidimensionality of these instruments.

#### Short versions of the WB-pro file

Researchers do not always have time to administer a 48-item instrument on well-being. Thus, Marsh and colleagues [1] developed two formative short versions of the WB-Pro, using a machine-learning algorithm. One included one item from each of the 15 WB-Pro factors, and one included the best five items. These short forms showed good psychometric properties and reproduced the pattern of associations with the correlates similar to the 48-item version of the WB-Pro. These short versions have good psychometric properties. However, they are suboptimal in comparison to the 48-item version of the WB-Pro in terms of profiling the multiple dimensions of well-being. Nonetheless, at times short versions might be useful. Therefore, we tested the two short versions of the WB-Pro for the Italian context. Both short versions are formative and thus provide a single global index of well-being instead of a profile of multidimensional factors.

#### A bifactorial representation of the WB-pro

Depending on the aim of an intervention or the specific research questions, it may be useful to have a single score of well-being. Although it is common practice, particularly among practitioners, to derive a single total score even from multidimensional instruments, this procedure should be done only after testing prerequisites for a composite score. Reise [28] noted that the presence of multidimensionality does not preclude the appropriateness of a total composite score. However, a global factor’s suitability should be evaluated with appropriate statistical procedures such as bifactorial models. In bifactor models, each item simultaneously reflects a global (G-factor) construct underlying responses to all items (global well-being), and specific (S-factors) components reflecting the variance shared among items forming a dimension but not explained by the G-factor [29]. The G-factor and all S-factors are orthogonal. The S-factors explain specific shared variance that links items of a component after controlling the variance explained by the G-factor. If the G-factor is sufficiently strong, it is appropriate to compute a total scale score.

This study aims to validate the WB-Pro in Italian as well as to better understand its multidimensionality through bifactor analysis.

## Method

### Participants

We collected data online through Google Forms (informed consent was acquired). Participants were recruited via announcements during university lessons and from the general population, through posts on social media. A total of 1451 participants (910 = women, 62.7%; age range: 18–70, M_age_ = 32.34, SD_age_ = 13.64) completed the survey online. The respondents were required to provide a personal code, composed of their birth year, the first two letters of their last name, and the first two letters of their first name. This code, matched with other demographic information, allowed us to check any potential double compilation.

The sample was composed of 170 (11.7%) individuals with no occupation, 580 (40.1%) students, 149 (10.3%) self-employers, 485 (33.5%) dependent workers, 23 (1.6%) retired and 41 (2.8%) other. Most participants lived in Sicily (79%), another 10% lived in Sardinia, and the remaining 11% resided in other Italian regions. Most participants had never been married (66.1%); 30.1% were married, and the remaining were divorced (2.6%) or widows (1%). Participants received no incentive for their involvement in the survey.

### Measures

#### Translation of the WB-pro file

The 48 items of the WB-Pro file were initially translated into Italian by a pool of three independent experts. Differences were discussed, and a final consensus was reached. An additional expert evaluated the translation, and the group adjusted some of the items after a collegial discussion. This version was back-translated into English and approved by a different independent expert.

#### Correlates of well-being and convergent/discriminant validity

We used several instruments to test convergent and discriminant validity. Concerning convergent validity, we expect overlapping or logically connected constructs to be associated to each other, showing high correlations. Therefore, to test convergent validity we used the Italian versions of the following instruments which include dimensions logically related to the subscales of the WB-Pro: Warwick Edinburgh Mental Well-being Scale (WEMWBS; [35, 22]), Flourishing Scale [36, 37], Psychological Need Satisfaction and Frustration Scale (PNSF- [38, 39]); we also examined life satisfaction, sleep quality and general health by translating into Italian respectively the standard life satisfaction questions from the U.K. Office for National Statistics [40], items from the Sleep Quality Scale [41], and a question adapted from the European Social Survey (ESS, [42]). All the items not already validated in Italian went through back translation procedures. In relation to discriminant validity, many studies have explored the relation between well-being and personality characteristics and found that traits and well-being are well-differentiated constructs, showing only moderate correlations (e.g., [30, 7, 31, 32]). Here we considered personality traits, measured with the Big Five Inventory (BFI- [33, 34]). Following Marsh and colleagues [1], we also included demographic correlates, such as gender, level of education, age, and marital status.

### Analyses

#### Confirmatory factor analysis versus exploratory structural equation modeling

Confirmatory Factor Analysis (CFA) and Exploratory Structural Equation Modeling (ESEM) models were estimated using Mplus 7.3 [43] robust Maximum Likelihood estimation. Models were estimated based on the full available information [44], to handle the few missing responses present at the item level (0 to 1.38%, M = 0.45%, SD = 0.34%).

When examining the factorial structure of a multidimensional instrument, such as the WB-Pro file, it is common that items present small associations with non-target factors. When CFA is used, factor correlations are likely to be positively biased due to the requirement of no cross-loading [45]. This does not happen with Exploratory Factor Analysis (EFA) models [46], but classic EFA models are not fully appropriate for confirmatory and predictive purposes. However, ESEM integrates EFA and CFA into a unified framework which provides the benefits of both techniques [47]. Specifically, target rotation [44] allows the specification of ESEM models in a confirmatory way by “targeting” all freely estimated cross-loadings to be as close to 0 as possible, but still allowing cross-loadings in multidimensional instruments, thus resulting in non-biased estimates. Therefore, we contrasted results from CFA and ESEM [44] to test the factor structure of the Italian version of the instrument^2^. We calculated composite reliability using McDonald’s [48] omega (ω) coefficient.

We conducted tests of measurement invariance across demographic groups (gender, age, and education) to test [49]: (a) configural invariance; (b) weak invariance (invariance of the factor loadings); (c) strong invariance (factor loadings, intercepts). These tests are used to ascertain if the measurement properties of a scale are equivalent across various groups (e.g., age, culture). Therefore, they are relevant to address mean differences across groups [49]. For example, if an instrument is invariant across age and we find that adolescents and adults have statistically different means, we can be sure that this difference pertains to groups’ characteristics and not to differences in the properties of the scale at different ages.

We evaluated the fit of all models with the following indices [50]: Comparative Fit Index (CFI), Tucker-Lewis Index (TLI), Root Mean Square Error of Approximation (RMSEA), and its 90% confidence interval. In relation to CFI and TLI, values greater than 0.90 and 0.95 indicate respectively adequate and excellent fit to the data; for the RMSEA, values smaller than 0.08 or 0.06 reflect acceptable and excellent model fit [50]. The chi-square test of exact fit (χ²) is also reported, even though it was always statistically significant given the large sample size. For the evaluation of nested models to assess invariance, since differences in χ^2^ are oversensitive to sample size and minor misspecifications we relayed on examinations of changes in fit indices [51]. For nested models, a CFI decline of 0.01 or less, and an RMSEA increase of 0.015 or less, indicate that the more parsimonious (i.e., invariant) model should be retained. Nevertheless, these proposed cutoff values are only rough guidelines [47].

#### Convergent and discriminant validity

We tested convergent and discriminant validity considering background variables and several psychological constructs (see the Measures section for details).

We also examined the relations between WB-Pro and two widely-used global measures of well-being purported to be unidimensional (Flourishing, WEMWBS – see Marsh et al., [1]). Here our focus was to determine whether these measures were unidimensional when factor analyzed with WB-Pro items. We followed Marsh and colleagues’ [1] procedure, using their *a priori* classification for categorizing Flourishing’s and WEMWBS’ items into different WB-Pro factors.

#### Bifactor analysis

Finally, to better understand WB-Pro’s multidimensionality, we also examined a bifactorial representation of the scale. Technical aspects to evaluate these models (e.g., fit indices) have already been discussed above.

## Results

### CFA versus ESEM

We begin with an evaluation of the WB-Pro factor structure based on CFA and ESEM models (see Table 1). Fit indices were reasonable for the CFA model (CFI = 0.932; TLI = 0.921; RMSEA = 0.042). However, they were substantially better for the ESEM model (CFI = 0.986; TLI = 0.968; RMSEA = 0.026). Thus, even though CFA is more parsimonious than ESEM, the fit indices controlling for parsimony (TLI and RMSEA) indicate that ESEM fits the data better, which is consistent with Marsh et al.’s [1] results.

**Table 1:** Fit indices for measurement models and invariance tests

			χ^2^	df	Scf	CFI	TLI	RMSEA
Models		CFA	3452.49	973	1.41	0.932	0.921	0.042
		ESEM	1029.70	511	1.32	0.986	0.968	0.026
		Bifactor-CFA	4253.59	101	1.42	0.910	0.903	0.046
		Bifactor-ESEM	979.60	478	1.21	0.986	0.967	0.027
Gender invariance		Configural	1966.05	1022	1.12	0.975	0.945	0.036
		Weak	2458.05	1517	1.28	0.975	0.963	0.029
		Strong	2526.87	1550	1.27	0.974	0.963	0.029
Education invariance		Configural	1899.95	1022	1.13	0.977	0.950	0.034
		Weak	2430.82	1517	1.26	0.976	0.965	0.029
		Strong	2494.73	1550	1.25	0.975	0.964	0.029
Age invariance		Configural	1891.69	1022	1.18	0.977	0.950	0.034
		Weak	2532.12	1517	1.28	0.973	0.961	0.030
		Strong	2627.07	1550	1.27	0.972	0.959	0.031

*Note*. χ^2^ values are all statistically significant for *p* < .001

The pattern of latent correlations among the 15 WB-Pro factors ranged from 0.130 to 0.898 (Mean r = 0.57) for the CFA model, and from 0.13 to 0.70 for ESEM (Mean r = 0.41, see Table S2 for details). In relation to factor loadings, both models were appropriate. In CFA, factor loadings ranged from 0.48 to 0.94. ESEM target factor loadings ranged from 0.30 to 0.98 (see Table S1 of supplemental materials for details) with the only exception of item 29^3^. Based on ESEM results we computed ω reliability scores for each scale (Autonomy = 0.93; Clear thinking = 0.90; Competence = 0.94; Emotional stability = 0.93; Empathy = 0.94; Engagement = 0.90; Meaning = 0.91; Optimism = 0.91; Positive emotions = 0.91; Positive relations = 0.94; Prosocial behavior = 0.92; Resiliency = 0.93; Self-acceptance = 0.91; Self-esteem = 0.94; Vitality = 0.91).

We also tested the invariance of the ESEM model over gender, age, and education. Results (Table 1) show that the fit indices do not decline substantially moving from configural to weak and strong invariance. Therefore, the structure of WB-Pro can be considered invariant over gender, age, and level of education, thus allowing mean comparison of different groups of individuals.

### Convergent and discriminant validity

We now evaluate support for the convergent and discriminant validity of WB-Pro latent factors in relation to relevant psychological constructs (Table 2). The patterns of associations are in line with theoretical expectations and similar to those found by Marsh and colleagues [1]. For example, the WB-Pro factors were positively related to the corresponding need satisfaction dimensions of the PNSF (0.60, 0.78, 0.44) and negatively related, and lower in size, to the corresponding need frustration dimensions (-0.41, − 0.56, − 0.32). In relation to personality traits, correlations of well-being are expected to be modest in size as personality traits only affect how one chooses to pursue potential and well-being [52]. Consistent with Marsh et al. [1], Big Five factors were modestly correlated with the WB-Pro factors (with the exclusion of some correlations *a priori* expected to be high, e.g., Emotional stability and Neuroticism; Agreeableness and Prosocial behavior), confirming the WB-Pro’s discriminant validity about personality factors. Finally, in relation to single-item measures, overall, the pattern of associations was close to those found by Marsh et al.’s study (1, see supplemental materials).

**Table 2:** Correlations between psychological constructs and 15 WB-pro factors

	C	E	N	O	A	SA	FA	SR	FR	SC	FC
Autonomy	**0.374**	**0.450**	**− 0.410**	**0.308**	**0.179**	**0.602**	**− 0.410**	**0.346**	**− 0.257**	**0.495**	**− 0.364**
Clear thinking	**0.625**	**0.397**	**− 0.430**	**0.355**	**0.295**	**0.545**	**− 0.254**	**0.371**	**− 0.246**	**0.669**	**− 0.448**
Competence	**0.471**	**0.268**	**− 0.134**	**0.324**	**0.238**	**0.341**	**− 0.207**	**0.281**	**− 0.235**	**0.436**	**− 0.316**
Emotional stability	**0.324**	**0.316**	**− 0.856**	**0.241**	**0.243**	**0.386**	**− 0.169**	**0.230**	**− 0.134**	**0.417**	**− 0.315**
Empathy	**0.315**	**0.297**	− 0.030	**0.312**	**0.655**	**0.345**	**− 0.092**	**0.488**	**− 0.126**	**0.296**	**− 0.104**
Engagement	**0.605**	**0.497**	**− 0.449**	**0.407**	**0.253**	**0.722**	**− 0.436**	**0.430**	**− 0.295**	**0.716**	**− 0.506**
Meaning	**0.555**	**0.459**	**− 0.381**	**0.330**	**0.245**	**0.666**	**− 0.338**	**0.482**	**− 0.323**	**0.633**	**− 0.478**
Optimism	**0.424**	**0.502**	**− 0.572**	**0.299**	**0.192**	**0.602**	**− 0.314**	**0.357**	**− 0.210**	**0.608**	**− 0.427**
Positive emotions	**0.462**	**0.627**	**− 0.536**	**0.337**	**0.344**	**0.617**	**− 0.401**	**0.527**	**− 0.393**	**0.568**	**− 0.463**
Positive relations	**0.327**	**0.320**	**− 0.149**	**0.251**	**0.337**	**0.430**	**− 0.365**	**0.777**	**− 0.561**	**0.356**	**− 0.329**
Prosocial behavior	**0.405**	**0.325**	− 0.079	**0.342**	**0.698**	**0.336**	**− 0.261**	**0.420**	**− 0.292**	**0.304**	**− 0.196**
Resilience	**0.374**	**0.447**	**− 0.657**	**0.301**	**0.110**	**0.403**	**− 0.192**	**0.262**	**− 0.209**	**0.484**	**− 0.355**
Self-acceptance	**0.459**	**0.503**	**− 0.573**	**0.351**	**0.317**	**0.553**	**− 0.267**	**0.441**	**− 0.309**	**0.615**	**− 0.493**
Self-esteem	**0.389**	**0.377**	**− 0.348**	**0.336**	**0.182**	**0.397**	**− 0.212**	**0.304**	**− 0.262**	**0.608**	**− 0.432**
Vitality	**0.458**	**0.604**	**− 0.196**	**0.160**	**0.240**	**0.513**	**− 0.291**	**0.376**	**− 0.220**	**0.514**	**− 0.369**

*Note*. C = Consciousness; E = Extraversion; N = Neuroticism; O = Openness; A = Agreeableness; SA = Satisfaction Autonomy; FA = Frustration Autonomy; SR = Satisfaction Relatedness; FR = Frustration Relatedness; SC = Satisfaction Competence; FC = Frustration Competence

In bold statistically significant correlations (*p* < .01)

**Links with other measures of well-being**. Here we evaluated how items from purportedly unidimensional measures of well-being (WEMWBS and the Flourishing) relate to different WB-Pro factors (see Table S4 of Supplemental materials). In the first model, the global factors for the Flourishing and WEMWBS were modelled as separate factors along with the 15 WB-Pro factors and the fit was modest (CFI = 0.901, TLI = 0.859, RMSEA = 0.047). In the second model, the Flourishing and WEMWBS items were allowed to load on the 15 WB-Pro factors, and the fit was substantially improved (CFI = 0.958, TLI = 0.930, RMSEA = 0.033). Consistent with Marsh et al.’s results this shows that WEMWBS and Flourishing instruments are not unidimensional. The pattern of loadings also supports the validity of the WB-Pro factors (see Supplemental Materials).

### Relations with demographic variables

A set of background variables was regressed on the 15 WB-Pro factors (see 1); for details see Table S3 in supplemental materials). Most of the background variables correlated positively with some WB-Pro factors but negatively with others. This demonstrates that the pattern of results could not be represented by a single global measure of well-being and supports the need for a multidimensional well-being measure. For example, men reported higher scores in Emotional stability, Self-acceptance, Optimism, and Vitality, whereas women had higher scores in Empathy, Positive emotions, and Meaning. Several WB-Pro factors increased with age (Empathy, Meaning, Self-acceptance, Resilience, and to a lesser extent, Emotional stability, and Clear thinking). However, other factors decreased with age (Prosocial behavior, Positive emotions, Optimism, and Autonomy). Individuals with higher educational levels showed higher scores on Emotional stability, Meaning, and Vitality, and lower scores on Optimism and Autonomy. Finally, being married resulted positively associated with some WB-Pro scales (e.g. Empathy r = 0.285), but negatively associated with other scales (e.g., Autonomy = − 0.205). These complex patterns of differences in WB-Pro factors would be lost if only a single global well-being factor was considered.

### Bifactor models of the WB-profile

We evaluated the bifactor models of the WB-Pro to better understand its multidimensional nature. Thus, even when the multidimensional factor is well-defined, it is reasonable to report a total score when the bifactor G-factor is sufficiently strong. However, if the subscales are sufficiently distinct, then reporting a total score is less defensible [28]. We tested CFA and ESEM bifactor models (Table 1). Again, the fit of the ESEM-bifactor model is better than the CFA-bifactor model (see Table 3 for factor loadings and reliability values of the bifactor-ESEM model).

**Table 3:** Factor loadings of G- and S-factors of WB-Pro

Item	G-factor	S-Aut	S-Think	S-Comp	S-ES	S-Emp	S-Eng	S-Mean	S-Opt	S-PE	S-PR	S-PB	S-Res	S-SA	S-SE	S-Vita	δ
WB7	**0.58**	**0.58**	0.01	0.09	0.03	0.06	0.00	0.04	0.00	0.05	0.03	0.07	0.11	0.07	0.05	0.06	0.020
WB12	**0.64**	**0.66**	0.02	0.08	0.04	0.10	0.05	0.03	0.04	0.02	0.01	0.02	0.02	0.02	0.03	0.03	0.019
WB23	**0.63**	**0.54**	0.03	0.04	0.03	0.07	0.07	0.01	0.01	0.06	0.01	0.02	0.00	0.09	0.01	0.03	0.019
WB29	**0.65**	0.07	**0.12**	0.03	0.05	0.12	0.10	0.03	0.07	0.05	0.05	0.05	0.02	0.10	0.11	0.00	0.022
WB36	**0.68**	0.01	**0.51**	0.14	0.00	0.02	0.10	0.04	0.03	0.01	0.04	0.01	0.01	0.04	0.07	0.01	0.018
WB46	**0.69**	0.02	**0.60**	0.12	0.06	0.02	0.07	0.00	0.03	0.01	0.04	0.01	0.02	0.01	0.01	0.00	0.016
WB5	**0.65**	0.05	0.10	**0.34**	0.11	0.09	0.01	0.04	0.01	0.10	0.02	0.02	0.03	0.03	0.10	0.04	0.021
WB17	**0.71**	0.01	0.10	**0.41**	0.04	0.04	0.16	0.03	0.01	0.02	0.07	0.01	0.02	0.01	0.21	0.02	0.014
WB18	**0.68**	0.01	0.10	**0.39**	0.06	0.05	0.22	0.03	0.01	0.02	0.06	0.05	0.01	0.03	0.20	0.03	0.015
WB10	**0.52**	0.05	0.01	0.07	**0.54**	0.03	0.02	0.09	0.04	0.04	0.06	0.04	0.04	0.03	0.04	0.05	0.021
WB39	**0.58**	0.01	0.07	0.07	**0.40**	0.15	0.07	0.08	0.02	0.06	0.05	0.02	0.15	0.15	0.03	0.02	0.021
WB44	**0.46**	0.06	0.02	0.02	**0.67**	0.01	0.00	0.07	0.07	0.03	0.11	0.09	0.04	0.08	0.01	0.01	0.022
WB4	**0.25**	0.09	0.06	0.07	0.16	**0.57**	0.01	0.05	0.06	0.06	0.04	0.08	0.10	0.08	0.07	0.03	0.028
WB24	**0.24**	0.05	0.07	0.10	0.11	**0.62**	0.01	0.06	0.06	0.04	0.03	0.04	0.08	0.05	0.08	0.02	0.028
WB41	**0.39**	0.05	0.01	0.03	0.00	**0.42**	0.01	0.02	0.05	0.05	0.05	0.31	0.06	0.01	0.11	0.09	0.028
WB43	**0.19**	0.04	0.01	0.02	0.09	**0.45**	0.01	0.01	0.03	0.07	0.06	0.14	0.00	0.06	0.04	0.03	0.027
WB13	**0.74**	0.07	0.09	0.07	0.02	0.05	**0.28**	0.07	0.01	0.05	0.03	0.06	0.00	0.04	0.08	0.08	0.015
WB19	**0.75**	0.02	0.01	0.25	0.01	0.03	**0.36**	0.05	0.08	0.13	0.01	0.01	0.00	0.01	0.13	0.05	0.015
WB35	**0.76**	0.03	0.15	0.08	0.01	0.01	**0.44**	0.10	0.03	0.01	0.05	0.03	0.02	0.01	0.01	0.01	0.014
WB2	**0.70**	0.14	0.11	0.00	0.10	0.10	0.01	**0.37**	0.04	0.02	0.08	0.09	0.09	0.15	0.02	0.09	0.021
WB33	**0.72**	0.01	0.06	0.00	0.03	0.01	0.18	**0.39**	0.14	0.03	0.01	0.02	0.03	0.10	0.08	0.01	0.016
WB38	**0.67**	0.06	0.11	0.09	0.06	0.09	0.05	**0.67**	0.17	0.10	0.02	0.04	0.04	0.01	0.09	0.07	0.017
WB3	**0.72**	0.03	0.07	0.02	0.05	0.13	0.02	0.14	**0.50**	0.05	0.00	0.12	0.03	0.07	0.02	0.01	0.015
WB11	**0.69**	0.04	0.05	0.01	0.14	0.05	0.03	0.06	**0.52**	0.08	0.08	0.08	0.12	0.03	0.03	0.09	0.014
WB45	**0.71**	0.03	0.05	0.00	0.05	0.01	0.09	0.14	**0.50**	0.14	0.02	0.03	0.04	0.05	0.06	0.06	0.015
WB27	**0.72**	0.02	0.04	0.01	0.02	0.07	0.02	0.03	0.06	**0.41**	0.10	0.02	0.07	0.01	0.02	0.21	0.015
WB42	**0.77**	0.04	0.01	0.03	0.02	0.04	0.06	0.05	0.10	**0.42**	0.06	0.04	0.01	0.04	0.08	0.04	0.012
WB48	**0.78**	0.03	0.02	0.03	0.05	0.11	0.01	0.02	0.12	**0.47**	0.01	0.04	0.04	0.01	0.05	0.10	0.012
WB1	**0.33**	0.06	0.06	0.01	0.08	0.00	0.07	0.02	0.07	0.01	**0.49**	0.04	0.09	0.10	0.12	0.08	0.027
WB20	**0.47**	0.07	0.00	0.04	0.00	0.03	0.04	0.06	0.03	0.07	**0.43**	0.01	0.08	0.02	0.02	0.01	0.025
WB26	**0.48**	0.01	0.03	0.01	0.05	0.06	0.04	0.00	0.01	0.08	**0.76**	0.06	0.02	0.03	0.01	0.01	0.024
WB28	**0.49**	0.02	0.00	0.08	0.08	0.03	0.00	0.05	0.07	0.02	**0.54**	0.06	0.07	0.02	0.14	0.04	0.025
WB31	**0.50**	0.04	0.00	0.02	0.11	0.18	0.02	0.05	0.09	0.06	0.05	**0.61**	0.10	0.00	0.02	0.05	0.024
WB37	**0.52**	0.04	0.05	0.05	0.04	0.20	0.02	0.01	0.04	0.03	0.05	**0.64**	0.08	0.01	0.04	0.03	0.021
WB40	**0.48**	0.00	0.00	0.01	0.01	0.19	0.03	0.04	0.10	0.01	0.07	**0.61**	0.10	0.02	0.01	0.06	0.024
WB6	**0.61**	0.01	0.01	0.03	0.05	0.06	0.03	0.03	0.04	0.04	0.05	0.12	**0.64**	0.07	0.02	0.06	0.017
WB9	**0.66**	0.05	0.02	0.02	0.04	0.09	0.01	0.01	0.00	0.01	0.10	0.10	**0.60**	0.05	0.01	0.08	0.017
WB34	**0.65**	0.02	0.05	0.05	0.14	0.09	0.06	0.03	0.09	0.07	0.08	0.07	**0.52**	0.13	0.02	0.05	0.016
WB14	**0.61**	0.11	0.02	0.02	0.18	0.08	0.02	0.04	0.13	0.01	0.10	0.07	0.19	**0.18**	0.00	0.02	0.019
WB21	**0.43**	0.01	0.02	0.03	0.01	0.05	0.02	0.08	0.04	0.08	0.12	0.12	0.06	**0.18**	0.10	0.03	0.027
WB22	**0.55**	0.03	0.07	0.02	0.02	0.02	0.03	0.00	0.06	0.02	0.01	0.04	0.04	**0.51**	0.04	0.01	0.022
WB32	**0.64**	0.05	0.04	0.04	0.05	0.07	0.01	0.06	0.03	0.08	0.04	0.05	0.04	**0.60**	0.00	0.02	0.021
WB15	**0.67**	0.00	0.09	0.21	0.05	0.04	0.01	0.08	0.02	0.04	0.00	0.05	0.05	0.05	**0.40**	0.00	0.019
WB16	**0.70**	0.00	0.04	0.18	0.00	0.08	0.02	0.02	0.01	0.08	0.00	0.05	0.00	0.04	**0.49**	0.00	0.016
WB47	**0.71**	0.08	0.16	0.11	0.03	0.05	0.07	0.06	0.02	0.02	0.01	0.03	0.00	0.05	**0.40**	0.04	0.017
WB8	**0.70**	0.08	0.05	0.03	0.02	0.08	0.02	0.03	0.01	0.07	0.06	0.08	0.07	0.04	0.01	**0.51**	0.015
WB25	**0.68**	0.01	0.02	0.01	0.02	0.03	0.05	0.02	0.08	0.11	0.06	0.04	0.05	0.01	0.05	**0.60**	0.016
WB30	**0.70**	0.03	0.04	0.01	0.00	0.06	0.08	0.02	0.07	0.17	0.03	0.02	0.07	0.04	0.01	**0.57**	0.014
ω	0.99	0.98	0.96	0.96	0.98	0.97	0.96	0.97	0.98	0.98	0.98	0.98	0.98	0.97	0.97	0.98	

*Note.* S-Aut = S-factor autonomy; S-think = S-factor think clear; S-Comp = S-factor competence; S-ES = S-factor emotional stability; S-Emp = S-factor empathy; S-Eng = S-factor engagement; S-Mean = S-factor meaning; S-Opt = S-factor optimism; S-PE = S-factor positive emotions; S-PR = S-factor positive relations; S-PB = S-factor prosocial behavior; S-Res = S-factor resiliency; S-SA = S-factor self-acceptance; S-SE = S-factor self-esteem; S-Vita = S-factor vitality. G-factor: construct underlying responses to all items (global well-being in our case); S-factors: components reflecting the variance shared among items after controlling the variance explained by the G-factor

Almost all items load 0.40 or higher on the general factor (Mdn = 0.65; Mean = 0.60; S.D. = 0.14). The only exception is three of the four Empathy items (which retain much specific variability, even after controlling for global well-being). This suggests that most items contribute substantially to the global factor of well-being, thus justifying the appropriateness of a total scale score.

Inspection of Table 3 suggests that for most of the 15 WB-Pro factors, there is a balance in the contribution to the global and specific factors, with factor loadings of at least 0.40 for most items on both specific and global factors (e.g., *autonomy*; *emotional stability*; *optimism*), but some items seem to contribute better to the global factor than to the specific factor (e.g., items 14 and 21 of the *self-acceptance* dimension).

### Short versions of the WB-pro file

We tested the two formative^4^ short versions developed by Marsh and colleagues [1] in the Italian sample. The patterns of associations between the short versions of the WB-Pro and the psychological correlates of well-being (Table 4) were comparable between the two short formative versions of the WB-Pro; however, the correlations for the 5-item version appeared to be slightly higher. We also compared correlations based on these short-form measures and global well-being based on the ESEM-bifactor G-factor (see Table 1 for fit indices and supplemental materials for additional information). The patterns of associations were similar across the short versions and the G-factor of well-being based on the 48-item version of the instrument (mean difference correlation of − 0.005 between the G-factor and the 15-item version, and of − 0.044 between the G-factor and the 5-item version), confirming the adequacy of the short forms proposed.

**Table 4:** Associations between psychological correlates and short versions of the WB-Pro

	WB-Pro 15	WB-Pro 5	G-factor	WB-Pro15 Factors	
S_Autonomy	0.77	0.77	0.71	Autonomy	0.60
F_Autonomy	− 0.43	− 0.43	− 0.39	Autonomy	− 0.41
S_Relatedness	0.58	0.65	0.60	Positive relations	0.78
F_Relatedness	− 0.40	− 0.44	− 0.39	Positive relations	− 0.56
S_Competence	0.80	0.83	0.71	Competence	0.44
F_Competence	− 0.58	− 0.59	− 0.52	Competence	− 0.32
BFC_consciousness	0.67	0.73	0.64	Competence	0.47
BFE_extraversion	0.68	0.79	0.62	Engagement	0.50
BFN_neuroticism	− 0.68	− 0.66	− 0.57	Emotional stability	− 0.86
BFA_agreeableness	0.42	0.55	0.48	Prosocial behavior	0.70
BFO_openness	0.48	0.57	0.48	Engagement	0.41
Flourishing	0.94	0.96	0.89		
WEMWBS	0.85	0.88	0.78		

*Note.* The versions WB-Pro 15 and WB-Pro 5 are based on the items identified by Marsh and colleagues [1]. The WB-Pro 15 factors are the same specific scales from the 48-item version of the WB-Pro used by Marsh et al. [1] for the validation of their two formative short forms. The G-factor has been extracted from the bifactor- ESEM that we computed on the 48-item Italian version of the WB-Pro.

## Discussion

Marsh and colleagues [1] developed the WB-Pro to measure multiple dimensions of well-being to balance a traditional focus on multiple components of mental illness. The 15 WB-Pro factors reflect both hedonic and eudaimonic components of well-being (e.g. [6, 56–58]). The detailed profile of different well-being factors provides more useful information for the evaluation of well-being interventions and clinical practice—reflecting relative strengths and weaknesses, that can be properly used to shape individualized interventions based on the limits and strengths of people and targeted groups.

The original WB-Pro showed a well-defined, multidimensional 15-factor structure with good psychometric properties and a solid theoretical background [1]. Therefore, it is useful to test this instrument in non-English-speaking countries. Here, we developed an Italian version of the instrument and tested it with a large Italian sample.

### CFA and ESEM

We tested the WB-Pro factor structure with CFA and ESEM. Consistent with Marsh and colleagues [1], CFA showed good factor loadings but inflated latent factor correlations and poorer fit indices compared to ESEM. The 15 WB-Pro factors were well-defined and reliable. The factors were differentially associated with various demographic variables supporting the multidimensionality of the instrument. For example, for age and gender, we found some positive relations and some negative relations with the WB-Pro factors. This result supports the importance of a multidimensional perspective measuring well-being (e.g. [1, 54, 56]).

Thus, the rich complexity of well-being differences in relation to background differences would be lost with a univariate perspective based on a single global score (e.g. [1, 15, 59]).

### Multidimensionality of WB-pro

WB-Pro’s multidimensionality was also supported by the differentiated pattern of correlations with psychological constructs used to test its convergent and discriminant validity. In line with expectations, for the basic psychological needs scale [39], the WB-Pro factors were more strongly and positively related to the corresponding need satisfaction dimensions and negatively and less strongly related to the corresponding need frustration dimensions. However, some of the PNSF scales showed also higher correlations with theoretically linked scales, different from the a-priori expectations (e.g., competence satisfaction more correlated with clear thinking than with competence). This might indicate a wider and more general content of the PNSF scales in comparison to the WB-Pro file scales, at least for the Italian versions. In line with theoretical expectations, Big Five factors showed only modest correlations with the WB-Pro factors [52]. Nevertheless, the largest correlations were for theoretically related factors (e.g., Extraversion with Vitality).

Significant correlations between the WB-Pro factors and other measures of well-being, life satisfaction, happiness, general health, sleep, and physical activity were consistent with *a priori* predictions (e.g., happiness with positive emotions and optimism; general health with vitality and positive emotions). Overall, the patterns of associations were similar to those found by Marsh et al. [1].

### Bifactor analysis

To further understand the multidimensional nature of the WB-Pro, we also tested a bifactor-ESEM model [28, 45]. The focus of this analysis was to evaluate the strength of the global well-being factor (i.e., the ESEM-bifactor G-factor). Almost all the WB-Pro items showed factor loadings on the general factor of 0.40 or more [55], suggesting that they all contribute to the definition of a general well-being factor. This supports the use of a total based on all 48 WB-Pro items. Interestingly, Empathy items had surprisingly small factor loadings on the global factor. Perhaps this is because empathic people often suffer from others’ miseries. Therefore, although clearly a relevant component of well-being, Empathy might also be associated with pain when others suffer or are in difficult situations. Nevertheless, it is important to replicate this result and, if generalizable, explore further the implications.

### Short formative forms

Finally, we tested in the Italian context the two formative short versions of the WB-Pro developed by Marsh and colleagues [1]. The 15-item and 5-item formative versions of WB-Pro were appropriate in the Italian context. However, they should be used only when it is not possible to administer the longer version of the WB-Pro, which remains a richer and more reliable instrument.

### Advantages and strengths of the WB-pro

Even though a few items of the Italian version of the WB-Pro might benefit from revision (e.g., *clear-thinking* scale), this study confirms the theoretical and empirical strength of the WB-Pro and its usefulness for applied psychology and clinical practice. From a theoretical point of view, it is interesting that the Huppert and So [21] model for the development of this instrument was based on the notion of well-being as positive mental health incorporating both hedonic and eudaimonic well-being. This supports the growing recognition of well-being as a complex profile of multiple components that cannot adequately represented by simplistic global measures.

The WB-Pro differs from most well-being instruments, particularly those representing well-being with one or a few global scores. Even among multidimensional instruments, few provide psychometrically strong measures with such broad coverage of different well-being constructs. WB-Pro’s multidimensionality is a defining characteristic important for applied interventions and clinical practice. For interventions, it is usually more useful to evaluate the intervention with a multidimensional profile of specific well-being factors rather than a global score. This provides better information about the nature of the intervention’s effects—its strengths and weaknesses. Similarly, for clinical practice, it is more useful to know a client’s specific strengths and weaknesses rather than a global assessment of well-being. Clinicians can then focus treatment on specific components of well-being that would not be possible if only global well-being were assessed.

WB-Pro’s multidimensional perspectives have a strong theoretical basis. The multiple components mainly parallel traditional diagnostic dimensions of mental illness. This design feature is likely to be useful to applied researchers and clinicians who also work with mental illness as well as mental health and well-being. However, for those who only want a global measure, there are two appropriate short versions of the WB-Pro. These short forms provide a formative global measure of well-being with a strong empirical basis. This is theoretically and empirically more appropriate than the typically reflective global score offered by most other instruments. Moreover, our bifactor analysis has shown that getting an appropriate global scale of well-being from the WB-Pro long version is possible.

Having such a broad tool composed of relatively few items makes it conceivable to imagine different solutions to affect the quality of life of people in our communities. Researchers and practitioners could test specific group and individual enhancement interventions based on each of the WB-Pro dimensions to improve well-being. Once the effectiveness of the enhancement interventions has been verified, they could be used in all places with value to people, e.g., at school, university, work, etc. The major impact we envisage is, as Seligman [4] and Huppert and Ruggeri [3] point out, that the assessment of well-being becomes the yardstick for constructing as well as the goal of public policies, rather than building them on mere increases in wealth or productivity. Improving the well-being of people, and even more so in a multidimensional way, can be the goal for the optimal functioning not only of individuals but of our communities of life.

## Limitations and recommendations for future investigation

The results of the study should be interpreted in relation to some limitations. The questionnaire was administered on a convenience sample, not balanced by socio-demographic variables (e.g., gender). Also, cultural influence may have influenced the results since the geographical origin (participants are mostly residents of the two islands of Italy) is not evenly distributed all over the country. Therefore, future studies should involve samples more representative of the population to further verify the factorial structure of the instrument with a more balanced sample. Moreover, we did not adopt specific measures in the survey to avoid response bias and to test the honesty of the participants. Future studies should involve control questions to avoid response bias and stimulate participants’ compliance.

Future research could use longitudinal research designs to test causal hypotheses more precisely, using, for example, dispositional traits and mediators and/or moderators influencing the WB-Pro dimensions. Moreover, future studies could confirm or disconfirm if our findings generalize to other cultural or national groups. We believe that some modifications could be made in future research to a few items (e.g., *clear thinking scale*) of the Italian version, given the different cultural context in which it was originally validated.

Finally, testing the instrument with clinical samples would be interesting for two reasons. First, to test the various forms of invariance on clinical and non-clinical samples. Second, longitudinal studies should be conducted to monitor progress and verify the effectiveness of psychotherapies. Additional hypotheses about the effects of different therapies could be tested as well, for example, therapies focused on constructing well-being (e.g. therapies centered on the theories and intervention methodologies of positive psychology) may increase well-being levels more in the areas of WB-Pro compared to other therapies.

Despite these limitations, the Italian version of WB-Pro provides some important advantages: researchers and practitioners can use it to understand the multidimensionality of well-being better and plan interventions to increase the specific areas contributing to well-being.

## Conclusion

The aim of the present study was to validate the WB-Pro in the Italian context, as well as to test the convergent and discriminant validity with other instruments evaluating well-being and its correlates. To address this aim, various instruments already validated in Italy were used. This study confirmed the 15-factor structure as well as the reliability and validity of the instrument. A bifactorial model was also tested in this study, and we found that almost all 48 items of the WB-Pro contribute to the definition of a general well-being factor. In addition, two reduced versions of the instrument, with 15 and 5 items, were also examined. We found these to be appropriate for the Italian context. However, given the theoretical premise of the multidimensionality of the instrument, they should only be used in cases where it is not possible to administer the 48-item version.

Despite the limitations, the Italian version of the WB-Pro offers some important advantages. The results of this study could be of interest to public health policies as they confirm the theoretical and empirical strength of the instrument, as well as its usefulness for psychological professional practice to support the evaluation of specific actions and interventions aimed at promoting positive well-being outcomes for the general population.

### Electronic supplementary material

Below is the link to the electronic supplementary material.

**Supplementary Material 1**: Italian Validation of the Well-Being Profile

## Data Availability

All materials and data are available from the first author via e-mail: lfscalas@unica.it.

## Footnotes

1 Based on Diener and colleagues work, Subjective well-being (SWB) is a specific term used to indicate the subjective evaluation of the quality of ones’ own life. Nonetheless, other authors consider SWB as a general term referring to the individual perspective on one’s well-being versus an objective perspective (e.g. the objective goods required to have a good life [53]).

2 It should be noted that, after randomizing the 48 items, we did not realize that a few items referring to the same construct were adjacent (items 17 and 18 of competence and items 21 and 22 of Self-Acceptance). Therefore, during the data analysis, it was appropriate to include a correlation between the error variance of these items in both CFA and ESEM models.

3 It should be noted that the problem of the low factor loading for item 29 (*Sono in grado di pensare con chiarezza* English: *I am able to think clearly*) on the Clear thinking scale is solved when items of WEMWBS and Flourishing are absorbed into the 15 WB-Pro file factors. Indeed, the saturation goes from 0.15 to 0.33. This might enlighten the initial low factor loading of item 29, it is possible indeed that items 36 and 46 (respectively *Sono in grado di concentrarmi facilmente quando è necessario* and *Sono in grado di rimanere concentrato quando ne ho bisogno* - English: *I am easily able to concentrate when necessary* and *I am able to stay focused when I need to*) are more strictly related to each other in terms of content in the Italian version and force the factor to a narrow meaning. Therefore, it might be useful in order to improve the Clear thinking scale (at least in the Italian version), to include an additional item with a content similar to WEMWBS item 8 *Ho pensato in modo chiaro* (English: *I’ve been thinking clearl**y*), which has a good factor loading on the clear thinking scale (0.404).

4 Instruments can be developed with a reflective or a formative approach (see 1). In the reflective approach the items are indicators of the construct and therefore express alternative versions of the construct. Each item is composed of a true variance (referable to the presence of the latent construct) and an error variance. Therefore, using multiple items protects from systematic errors. Moreover, even if we eliminate one of the items, the latent construct will still be defined by the other alternative items (net of measurement errors). The alternative items will be highly correlated showing internal consistency. In the formative approach, on the contrary, the items express different aspects of the construct and therefore, serve to define the construct itself. If one item is eliminated, the meaning and nature of the construct will change. Therefore, items are not necessarily correlated to each other and are not required to be internally consistent. Nonetheless, they provide a synthetic index that embraces various aspects forming the construct under examination.
