# Supplementary material for: Towards a multidimensional measure of well-being: cross-cultural support through the Italian validation of the well-being profile
Source: BMC Psychol. 2023 Dec 13;11:441. doi: 10.1186/s40359-023-01485-9 (PMC10720179; doi:10.1186/s40359-023-01485-9)
Supplement: Supplementary file 1 — Supplementary Material 1: Italian Validation of the Well-Being Profile [file 40359_2023_1485_MOESM1_ESM.docx]

**Supplemental materials of the paper: Towards a multidimensional measure of well-being: Cross-cultural support through the Italian validation of the Well-being Profile**

**The most popular measures of well-being**

There is an increasing interest in measuring well-being, not only by an individual perspective, but also by a collective one: in fact, measuring well-being helps to monitor a nation’s “happiness” and the efficacy of policy decisions taken to improve the well-being of a nation. For example, the United Nations Sustainable Development Solutions Network (SDSN) publishes annually the World Happiness Report that ranks countries by how “happy” they are (Helliwell et al., 2020). Moreover, the WHOQOL-100 (Skevington, 1999) and WHOQOL-BREF (WHO, 1996) have been published by the World Health Organization (WHO) as measures of quality of life and well-being. Therefore, a single, standardized definition of well-being cannot be assumed in the literature (Ong et al., 2021). Nevertheless, it is important to consider how well-being is conceptualized in research, as from its conceptualization derives the construction of measuring instruments. Some instruments investigating well-being are unidimensional and focus on some narrow aspect of well-being or move from a quite specific definition of well-being, other instruments derive from a more complex vision of well-being, thus including several domains or components, nonetheless quite often they provide a single total score thus losing relevant and useful information (see Marsh et al. 2020, for a more detailed discussion on this topic).

The Personal Well-being Index (PWI; Cummins et al., 2003) is a generic measure of SWB; it uses an 11-point, end-defined Likert scale (ranging from 0, extremely dissatisfied, to 10, extremely satisfied with and a mid-point labelled ‘neither satisfied nor dissatisfied’). The PWI asks how satisfied people are with seven life domains: standard of living, personal health, achievement in life, personal relationships, personal safety, community-connectedness, and future security (Lau, Cummins, & McPherson., 2005). Several studies have demonstrated its use as a cross-cultural measure of subjective well-being, in Hong Kong and Australia (Lau et al., 2005), across Australia, Bosnia and Herzegovina, Croatia, and Serbia (Jovanović, Cummins, Weinberg, Kaliterna, & Prizmic-Larsen, 2019). Moreover, the PWI has been validated on Chilean adults (Gallardo-Peralta et al., 2019), and Indian adults (McIntyre, Saliba, & McKenzie, 2020), confirming the good psychometric properties.

The Satisfaction with Life Scale (SWLS; Diener, Emmons, Larsen, & Griffin, 1985; Pavot & Diener, 2009) was developed to assess global life satisfaction. Life satisfaction refers to a judgmental process, in which individuals assess the quality of their lives basing of their own unique set of criteria (Shin & Johnson, 1978). As reported by Pavot & Diener (2009), the SWLS shows good convergent validity with other scales and with other types of assessments of subjective well-being. The SWLS scores for life satisfaction show a good temporal stability, so that the SWLS could be considered as sufficiently sensitive to detect changes in life satisfaction during clinical intervention. Moreover, the scale can discriminate life satisfaction from emotional well-being. It is the most widely used scale, as it has shown excellent psychometric properties despite its shortness (5 items with a 7-point Likert scale) and it has been adapted in most cultural context all over the word: in Europe – i.e., Germany (Glaesmer, Grande, Braehler, & Roth, 2011), Sweden (Hultell & Gustavsson, 2008), Norway (Clench-Aas, Nes, Dalgard, & Aarø, 2011), Spain (Vàzquez, Duque, & Hervas, 2013), Italy (Di Fabio, & Gori, 2016; Di Fabio, & Gori, 2020); in South America – i.e., Brazil (Gouveia, Milfont, Da Fonseca, & de Miranda Coelho, 2009), Chile (Vera-Villarroel, Urzúa, Celis-Atenas, & Silva, 2012); in Asia - i.e., Palestina (Abdallah, 1998), Iran (Bayani, Koocheky, & Goodarzi, 2007), Malaysia (Swami & Chamorro-Premuzic, 2009), Hong Kong (Sachs, 2003).

The PANAS is a 20-item scale that express emotions: 10 evaluate negative affect (NA), the other 10 evaluate positive affect (PA). The PANAS focuses on the affective dimension of subjective well-being; it has been developed in the USA context (Watson et al., 1988); the respondents are asked to indicate the extent to which they experienced each of 20 emotions, reflecting PA and the remaining 10 reflecting NA. The literature review provides several adaptations in diverse cultural and linguistic contexts: Spanish (Sandín et al., 1999), adults’ UK population (Crawford & Henry, 2004), Estonian (Allik & Realo, 1997), German (Krohne, Egloff, Kohlmann, & Tausch 1996), Russian (Balatsky & Diener, 1993), Swedish (Hilleras et al., 1998), Turkish (Gencoz, 2000), Italian (Terracciano, McCrae, & Costa Jr., 2003), and Mexican (Robles & Páez, 2003). All the cited studies show good psychometric properties of the scale across the different samples.

The WHO-5 is the 5-item World Health Organization Well-Being Index, a short and generic global rating scale measuring subjective well-being; it is a frequently used brief standard measure in large-scale cross-cultural clinical studies. The WHO-5 has been used extensively worldwide: Topp and colleagues (2015), in their systematic review of the literature on the WHO-5, have recognized the different regions that have applied the scale: Africa (Algeria, South Africa), Asia (Bangladesh, China, India, Japan, South Korea, Sri Lanka, Taiwan, Thailand), Europe (Northern, Southern, Eastern, Western and Central Europe), the Americas (Canada, the US, Brazil, Mexico), the Middle East (Israel, Iran, Lebanon) and Oceania (Australia, New Zealand).

The second group of instruments is composed of scales that measure eudaimonic well-being: the PERMA-profiler (Butler, & Kern, 2016), the Psychological Well-being Scale (PWBS; Ryff & Keyes, 1995), the Flourishing Scale (FS; Diener, Wirtz, Tov, Kim-Prieto, Choi, Oishi, & Biswas-Diener, 2010), and the Basic Psychological Need Satisfaction and Frustration Scale (BPNSFS; Chen, Vansteenkiste, Beyers, Boone, Deci, et al., 2015) The PERMA-profiler is a 23-items scale based on the PERMA model (Seligman, 2011), evaluated on a 10-point Likert scale. It is composed of five dimensions (positive emotions, engagement, relationship, meaning, and accomplishment), with three items each, and eight additional items assessing: overall wellbeing (one item); sadness, anger, and anxiety (three items); loneliness (one item); and self-perceived physical health (three items) (Butler & Kern, 2016). It has been validated in Greece (Pezirkianidis, Stalikas, Lakioti, & Yotsidi, 2021), Japan (Watanabe, Kawakami, Shiotani, Adachi, Matsumoto, et al., 2018), Italy (Giangrasso, 2021), Australia (Ryan, Curtis, Olds, Edney, Vandelanotte et al., 2019), Germany (Wammerl, Jaunig, Mairunteregger, & Streit, 2019).

The PWBS (Ryff & Keyes, 1995) in its original form consisted of 20 items per scale and includes a total of 120 items. Shortened versions of the PWBS containing 84 items (14 items per scale), 54 items (9 per items per scale), 42 items (7 items per scale) and 18 items (3 items per scale) have also been used in research (Abbott et al., 2006). Van Dierendonck (2004) developed an alternative short version of the PWBS consisting of 39 items (Henn, Hill, & Jorgensen, 2016). Various versions of the Ryff’s scales were culturally adapted and tested in some European cultures, such as Serbia (Nišević & Cigić, 2013), Spain (Freire, Ferradas, Núñez, & Valle, 2017), Romania (Costea-Bărluțiu, Bălaș-Baconschi, & Hathazi, 2018), and Italy (Sirigatti, Penzo, Iani, Mazzeschi, Hatalskaja, et al., 2013), as well as some Asian cultures, as Korea (Choi & Choi, 2016), China and Taiwan (Li, et al., 2014), and some English-speaking cultures, such as the U.S.A (Hsu, Hsu, Lee, & Wolf, 2017) and Australia (Burns & Machin, 2009).

The BPNSFS is grounded in Basic Psychological Needs Theory (BPNT; Deci & Ryan, 2000; Ryan & Deci, 2000); it is a 24-item scale that consists of six 4-item subscales assessing Autonomy satisfaction, Competence satisfaction, Relatedness satisfaction, Autonomy frustration, Competence frustration, and Relatedness frustration. In addition to the original study conducted by Chen et al. (2015), that have validated the scale across four different cultural contexts (Belgium, China, USA, and Peru), the BPNSFS has been validated in Japanese (Nishimura & Suzuki, 2016), Polish (Kuźma, Szulawski Vansteenkiste, & Cantarero, 2020), German (Heissel, Pietrek, Flunger, Fydrich, Rapp, et al., 2019), and Italian (Costa, Ingoglia, Inguglia, Liga, Lo Coco, & Larcan, 2018; Liga, Ingoglia, Cuzzocrea, Inguglia, Costa, et al., 2018).

The FS (Diener et al., 2010) is an 8-item scale designed to measure social–psychological prosperity; it includes items on social relationships, on having a purposeful and meaningful life, on being engaged and interested in one’s activities, on self-respect and optimism, and, finally, on feeling competent and capable in the activities that are important to the respondent. Thus, the brief scale assesses major aspects of social–psychological functioning from the respondent’s own point of view. Although multidimensional in nature, this scale is used as a unidimensional one providing a single total score in which relevant information is lost. The FS is largely used because of its shortness and has been adapted in many diverse cultural contexts, as New Zealand (Hone, Jarden, & Schofield, 2014), Portugal (Silva & Caetano, 2013), Japan (Sumi, 2014), India (Singh, Junnarkar, & Jaswal, 2016), China (Tang, Duan, Wang, & Liu, 2016), France (Villieux, Sovet, Jung, & Guilbert, 2016), and Italy (Giuntoli, Ceccarini, Sica, & Caudek, 2017).

The traditional distinction between hedonic and eudaimonic perspectives of well-being is represented in some multidimensional instruments incorporating both perspectives. For example, the Hedonic and Eudaimonic Motives for Activities scale (Huta and Ryan, 2010) provides two factors: hedonia and eudaimonia. However, as noted by the authors, their scale evaluates the motives that lead people to follow activities rooted in hedonia or eudaimonia. Thus, hedonia and eudaimonia refer to motives for acting and represent independent variables that function as predictors of well-being, not two forms of well-being. Another instrument that includes both hedonic and eudaimonic aspects is the Euthymia scale (Fava, Bech, 2016). However, this 10-item instrument, partially derived from the WHO-5, is based on a true-false answer format. It forces respondents to choose between only two options, thus reducing variability in answers and determining potential biases. For this reason, Carrozzino et al. (2021) developed a 6-point Likert scale. However, the Euthymia scale provides only a single total indicator, thus losing valuable information.

The limitation also applies to the Warwick Edinburgh Mental Well-being Scale (WEMWBS; Tennant, Hiller, Fishwick, Platt, Joseph, et al., 2007).The WEMWBS is a 14-items self-reported scale covering both hedonic and eudaimonic aspects of mental health including positive affect (feelings of optimism, cheerfulness, relaxation), satisfying interpersonal relationships and positive functioning (energy, clear thinking, self-acceptance, personal development, competence, and autonomy) (Tennant et al., 2007). As it was for the FS, also the WEMWBS can be considered multidimensional in nature, but is treated as a unidimensional scale and provides only a total score of well-being. It has been developed and validated in the UK context; subsequently it has been adapted in very different linguistic and cultural contexts: Indonesian (Wicaksono, Roebianto, & Sumintono, 2018), Spanish (Lopez, Gabilondo, Codony, Garcia-Forero, Vilagut, et al., 2013), Chile (Carvajal, Aboaja, & Alvarado, 2015), Mexican (Hoffman, Rueda, & Lambert, 2019), Polish (Konaszewski, Niesiobędzka, & Surzykiewicz., 2021), Iranian (Mavali, Mahmoodi, Sarbakhsh, & Shaghaghi, 2020), showing good psychometric properties in all the studies.

Finally, the Mental Health Continuum—Short Form (MHC-SF) (Keyes, 2002) measures the three dimensions of hedonic well-being (positive affect and life satisfaction), social well-being (social acceptance, social actualization, social contribution, social coherence and social integration) and psychological well-being (autonomy, environmental mastery, personal growth, positive relations with others, purpose in life and self-acceptance). It has been validated in many different cultural contexts, such as Holland (Lamers, Westerhof, Bohlmeijer, ten Klooster, & Keyes, 2011), Italy (Petrillo, Capone, Caso, & Keyes, 2015), Poland ((Karaś, Cieciuch, & Keyes, 2014), Spain (Echeverría Errázuriz, Torres Sahli, Pedrals Gibbons, Padilla Pérez, Rigotti Rivera, & Britan Carreño, 2017), and in several cross-cultural studies (Joshanloo, Wissing, Khumalo, & Lamers, 2013; Żemojtel‐Piotrowska, Piotrowski, Osin, Cieciuch, Adams, et al., 2018; Rahkman Ardi, 2018), providing evidence of good psychometric properties.

**Description of the dimensions measured with the Wb-Profile**

In the following description of the literature review on the 15 dimensions of the WB-Profile, the studies have been grouped even though researchers have often adopted different perspectives to study well-being. For example, a specific dimension, depending on the perspective adopted by researchers, can be considered a predictor or correlate of well-being in one study, whereas it can be considered as a constituent element of well-being in other studies.

**Competence**

In literature there are many definitions of competence based on the perspective with which we look at human being and his/her interaction with the environment. Most of the definitions seem to imply that competence could be defined as the ability to do something effectively responding to individual and or contextual demands. From the perspective of well-being, the need for competence appears as one of the three basic psychological needs (Ryan & Deci, 2001) and it has been studied in valued contexts (for example in work and educational context) as a dimension related to well-being. For example, a study in this framework has highlighted that satisfaction of the needs for competence is related to higher levels of job satisfaction (Hofer & Busch, 2011). As Marsh and colleagues (2020) reported, a high sense of general competence is related to higher self-esteem (Thøgersen-Ntoumani & Ntoumanis, 2007) and satisfaction with life (Meyer, Enstrom, Harstveit, Bowles, & Beevers, 2007), instead a lack of general competence is related to anxiety and depression (Ryan & Deci, 2017; Wei, Shaffer, Young, Zakalik & Hansen, 2005).

In a socio-cognitive perspective, feeling competent at the tasks is essential for successful performance (Bandura 1997), anticipates the receiving of favourable outcomes (Carver & Scheier 2002) and improves domain specific satisfaction in different educational levels (Lodi et al., 2019; Magnano et al., 2020). Other types of competencies that do not strictly concern the performance of an educational or work task, such as for example psychological, personal and social competences affect PWB and are related to less distress perception (Fotiadis, Abdulrahman, & Spyridou, 2019; Holopainen, Lappalainen, Junttila, & Savolainen, 2012; Griffin, Botvin, Scheier, Epstein, & Doyle 2002).

**Clear thinking**

Clear thinking is a construct associated to competence and concerns the ability to think, concentrate, and make decisions (Marsh et al., 2020). Several studies have demonstrated that there is a positive relation between higher problem-solving and decision-making skills and well-being (Cenkseven-Ouml, 2013; Chang et al., 2009). Conversely, many research studies conclude that deficiencies in problem solving and decision-making skills are related with suicidal ideation (Siu and Shek, 2010), anxiety (Belzer et al., 2002; Nezu et al., 2004) and depressive symptoms (Nezu et al., 2004; Siu and Shek, 2010), which all indicate weakness in the individual's well-being (Chang et al., 2009).

**Emotional stability**

Emotional stability has often been studied as a personality trait in relation to perceived levels of well-being and refers to predictability and consistency in emotional reactions with an absence of rapid mood changes. It is related to SWB (positive and negative affect, life satisfaction) in various age groups, often playing a role of a strong predictor on wellbeing indexes (Brajša-Žganec, Ivanović, & Lipovčan, 2011; Suar, Jha, Das, & Alat, 2019; Butkovic, 2012; Morris, Burns, Periard, & Shoda, 2015). In a recent study, emotional stability was found as a predictor, among other personality traits, of life satisfaction and positive affect, and the only variable that negatively predicted the levels of negative affect (Kobylińska, Zajenkowski, Lewczuk, Jankowski, & Marchlewska, 2020). In a cross-cultural study, emotional stability was an important predictor of SWB in Mozambican, USA, and Portuguese sample (Galinha, Oishi, Pereira, Wirtz, & Esteves, 2013). Finally, a recent meta-analysis (Houben, Van Den Noortgate, & Kuppens, 2015) showed that positive emotional functioning, also referred to as stability of emotions, is also related to PWB and flourishing.

**Engagement**

According to Seligman (2011), engagement refers to the optimal experience, the flow, what gives rise to a "life full of involvement". It presupposes the use of the greatest strengths and talents that lead to the loss of self-awareness and sense of time. Some studies have focused on the role of engagement in places that are highly meaningful to people (such as at school and at university) with PWB and SWB levels, underlining the relationship between educational engagement, educational achievement, and well-being (Heffner & Antaramian, 2016; Antaramian, 2015; Orkibi & Tuaf, 2017; Gutiérrez & Tomás, 2019). Findings from several studies suggest a bidirectional relationship between school engagement and some indices of well-being such as life satisfaction (Lewis & Huebner, 2011), even though Datu & King (2018), through a longitudinal study, highlighted the predictive role of life satisfaction on school engagement. In a recent study, Luruli and colleagues (2020), for example, have found that engagement mediated the relation between study demands, study resources and well-being. The same line of research was used in the workplace, looking for the links between work engagement, work performance and perceived well-being. Literature showed that living higher level of work engagement is related with work performance, greater levels of life satisfaction, job satisfaction and health well-being (Junça-Silva, Caetano, & Lopes, 2017; Ahmed, Rehman, Ali, Ali, & Anwar, 2018; Yan, Su, Wen, & Luo, 2019; Bal, 2020; Lizano 2021). Finally, well-being is for example a crucial outcome of engagement in entrepreneurship (Nikolaev, Boudreaux, & Wood, 2017). The studies on engagement and well-being have not only concerned the "productive" contexts: important contributions are provided by the contexts of people's free time, their hobbies, and their interests outside of educational or work contexts. For example, a study has shown that engagement in leisure time has shown strong associations with SWB levels (Schulz, Schulte, Raube, Disouky, & Kandler, 2018).

**Meaning**

The dimension of meaning is one of the five components of Seligman's Perma model: according to the author, a meaningful life is characterized by having beliefs in something greater, that transcend our individual life and subjective states, and are related to higher purposes: for example, religion, politics, ecology, family, associations, values, feeling to belong to a community, feeling a mission in life. According to this perspective, human beings are looking for a meaning or a purpose in life, for everything that makes "a life full of meaning" and this increases people wellbeing. In this field, studying the role of pleasure, engagement and meaning in a very large sample, Vella-Brodrick, Park and Peterson (2009) found that meaning and engagement were the most important predictors of subjective well-being (satisfaction with life, positive affect and negative affect), also taking into account personality traits and sociodemographic variables. Moreover, having goals or purposes is also part of the constitutive dimension of well-being in both the socio-cognitive theory of well-being and Ryff's PWB model. The presence of meaning in life is related to life satisfaction, subjective happiness and PWB (Grozdanovska, 2016; Damásio & Koller, 2015; Kállay, 2015; Krok & Telka, 2019). A meta-analysis conducted by Roepke, Jayawickreme and Riffle 2014) demonstrated that levels of meaning are associated with physical health and with factors that promote positive health outcomes. Several studies have studied the role of meaning in life as mediator: Aglozo and colleagues (2021) highlighted that spirituality affected indirectly SWB through optimism and meaning in life and Stănculescu (2016) showed that self-esteem and optimism can contribute to the presence of meaning in life which in turn facilitates the perception of SWB.

**Optimism**

Optimism can be defined as the propensity to learn from experience and build positive future scenarios (Seligman et al., 2005). Optimism or pessimism refers to the way in which the causes and implications of events are explained in terms of internality. Optimism is linked to having a better level of physical health, quality of life, psychological well-being, hope, resilience, self-esteem, social skills, positive perceptions about the present and the future; optimistic individuals have better social relationships, are generally perceived more positively by others, and are more capable of reacting to stressful events; moreover, optimism is related to lower levels of anxiety, depression, maladaptive behaviours, victimization and social exclusion (Alloy et al., 2006; Ben-Zur, 2003; Deptula et al., 2006; Lemola et al., 2011; Reivich et al., 2013). Many studies founded optimism related to SWB and PWB in various age group (Duy & Yıldız. 2019; Krok & Telka, 2019; He, Cao, Feng, Guan, & Peng, 2013; Stănculescu, 2016; Goodarzi, et al., 2015; Heinitz, Lorenz, Schulze, & Schorlemmer, 2018) and in particular Ferguson and Goodwin (2010) stated that optimism was a strong predictor of both subjective and psychological well-being; on the contrary, pessimism was related to depressive symptoms, negative affect and stress (Landa et al., 2011).

**Positive emotions**

Positive emotion is another dimension of PERMA model (Seligman, 2011). Positive and negative emotions affect SWB through the frequency of positive and negative emotional states. If the events that happen in our various life contexts are experienced as positive, then they will produce positive emotions (joy, happiness, etc.). Conversely, if they are perceived as negative and stressful, then they will produce negative emotions (pain, anger, etc.) that will tend to prevail in our emotional experience (Zambianchi, 2015). Positive affectivity is related to other positive psychology construct such as hope, instead negative affectivity could be an obstacle to live positive experiences (Ng, 2017). The quality, intensity, and frequency of perception of positive and negative emotions is strongly affected by dispositional factors as the personality traits (Ng, 2015), especially neuroticism (Ng, Russell Kua, & Kang, 2019). Díaz and Arroyo (2013) showed that extroversion and neuroticism affected directly and significantly on positive and negative affect and indirectly on satisfaction with life. Finally, a longitudinal study (Li et al., 2014) has demonstrated that positive and negative emotions are two dimensions more stable than life satisfaction.

**Positive relations**

Positive relations characterize individuals who have warm, satisfying, trusting relationships with others (Ryff & Keyes, 1995); in other words, this dimension is referred to the depth of connection individuals have in ties with significant others (Ryff, 2013). A literature review, analysing over 18,000 articles, has highlighted the strong connection between social relationships and health (Tay, Tan, Diener, & Gonzalez, 2012). The relational dimension has a well-established place in most of the major existing wellbeing surveys used internationally (Butler & Kern, 2016). The literature review conducted by Pezirkianidis and colleagues (2021), indicates that high levels of the main function of positive relationships correlates to high levels of wellbeing and life satisfaction (Chandoevwit & Thampanishvong 2016); significant relationships are associated to the experience of positive emotions, give meaning in individuals’ lives, and promote psychological and physical health (Bryant & Veroff, 2007; Chandoevwit & Thampanishvong, 2016; Chopik, 2017). Positive relationships not only boost the positive elements of one’s life but also reduce negative components as well, e.g., stress, distress, loneliness, and depression (Caron & Liu 2011; Carmichael et al. 2015).

**Resilience**

Resilience can be defined as the ability to engage and persist even in the presence of failures and particularly negative events (Roberts, Brown, Johnson, & Reinke, 2002). It may consist of a response to psychological tension related to undesirable experiences (Tugade & Fredrickson, 2004) and a protective factor to cope with risks (Di Maggio et al., 2016). Seligman (2011) reports the distinction between recovery (the return to pre-traumatic levels of functioning after manifesting significant symptoms) and resilience (the ability to maintain a stable balance in the face of adverse events), defining resilience as an underestimated phenomenon, more common than recovery. Therefore, for the author, resilience is what allows human beings more probably to grow through adversity rather than succumb to it. The literature showed that resilience is linked to having a better level of self-efficacy, optimism, courage, self-esteem, problem solving and decision-making skills. People with high levels of resilience show greater propensity to think and plan their future, cope better with negative emotions, adapt better to their own contexts, and show greater ability to request support from others (Fredrickson, 2001; Lundman et al., 2007; Masten, Tellegen, 2012; Reivich et al., 2013). Finally, resilience is related with SWB in different sample: university students, workers, vulnerable persons (Zubair, Kamal, & Artemeva, 2018; Rani, 2019; Nartova-Bochaver, Donat, & Rüprich, 2019; Burns, Anstey, & Windsor 2011; Khan, 2013; Bajaj & Pande, 2016; Lau, Chiesi, & Saklofske, 2019; Nalin & de Freitas Pinho França, 2015; He et al., 2013; Bhattarai, Jin, Smedema, Cadel, & Baniya, 2021; Satici, 2016).

**Self-esteem and Self-acceptance**

Self-acceptance indicates a positive attitude toward the self, acknowledging and accepting both good and bad qualities and feeling positive about past life (Ryff & Keyes, 1995); it is also defined as the knowledge and acceptance people have of themselves, including awareness of personal limitations (Ryff, 2013). MacInnes (2006) found that an increase in levels of self-esteem and self-acceptance would be associated with a positive effect on the psychological well-being; according to his results, very strong appears the relationship between the increase in unconditional self-acceptance and well-being. Other studies, then, highlighted that self-acceptance is strongly inversely related to anxiety symptoms, whilst it is significantly positively correlated with happiness and satisfaction with life (Chamberlain & Haaga, 2001). Paradise and Kernis (2002) in their study, provided evidence that high and stable self-esteem is associated with several important components of positive psychological functioning, including self-acceptance.

**Vitality**

The term vitality is based on self-determination theory (Deci & Ryan, 2000) and is defined as the state of feeling alive and alert (Ryan & Deci, 2001) or having physical and mental energy (Ryan et al., 2010). In other words, Ryan e Frederick (1997) reported that psychological energy – defined as vitality – reflects well-being and supports the healthy lifestyle, increasing the sense of control. Govindji and Linley (2007) showed that vitality was significantly and strongly correlated with subjective well-being. Individuals with higher vitality tend to have autonomous initiatives and to rely on their own motivations to act; subsequently, these individuals are happier and satisfied of their lives, as they perceive higher levels of initiative and autonomy (Rodriguez-Carvajal, Moreno-Jiménez, & van Dierendonck, 2010)

**Autonomy**

The construct of autonomy is one of the dimensions that appears in the Ryff’s model of PWB. Autonomy can be defined as the capacity of people to make independent voluntary decision, to operate autonomously of external influences by using their judgement. Autonomy can support individuals’ success in many domains, it is a strong predictor of life satisfaction and happiness (SWB) practically in every age group and roles (e.g., students, workers, etc.) (Sheldon, Kasser, Houser-Marko, Jones, & Turban, 2005; O’Donnell, Chang, Miller, & Ma, 2013; Ng, 2015; Fotiadis, et al. 2019; Ng et al. 2019); conversely, a reduction of personal autonomy can have a detrimental effect on PWB (Kachanoff, 2019). In a study on a large sample, Olesen, Thomsen and O’Toole (2015) showed that a higher level of autonomy orientation predicted increased SWB above the personality traits of neuroticism and extraversion. A recent metanalysis (Shi Yu, Levesque-Bristol, & Maeda, 2018) highlighted that the satisfaction of basic need for autonomy is related to well-being, in particular to SWB, and no differences were founded in non-Western cultures compared with Western cultures about the hypothesis of a lower valuation of the individual autonomy in the collectivistic cultures*.*

**Empathy**

Empathy is broadly defined as “a set of constructs that connects the responses of one individual to the experiences of another. These constructs specifically include both the processes taking place within the observer and the affective and non-affective outcomes that result from those processes” (Davis, 2006, p. 443). Empathy is essential to positive social functioning (Batson, 1991; Eisenberg et al., 2007), and many research studies have found that empathy and well-being are related (Bourgault, Lavoie, Paul-Savoie, Grégoire, Michaud, et al., 2015; Cho & Jeon, 2019), as empathy is a predictor of subjective well-being in students (Cañero Pérez, Mónaco Gerónimo, & Montoya Castilla, 2019) and adults (Bourgault et al., 2015).

**Prosocial behaviour**

Prosocial behaviour is defined as “voluntary behaviour intended to benefit another” (Eisenberg et al., 2007, p. 646). It is an umbrella term encompassing actions to benefit others (Dovidio, Piliavin, Schroeder, & Penner, 2006). Prosocial behaviour is related to empathy but conceptually distinct from it, as prosocial behaviour describes observable behaviour, whereas empathy is referred to an internal state (Marsh et al., 2020). The relationship between prosocial behaviour and well-being has deeply explored through empirical studies (e.g., Aknin et al. 2013; Poulin et al., 2012), both with cross-sectional designs (see, i.e., the review conducted by Piliavin 2003), and with experimental manipulation; the overall results suggest that prosocial behaviour leads to increased well-being (e.g., Weinstein & Ryan, 2010; Williamson & Clark, 1989). Moreover, in a more recent works, Martela and Ryan (2016) and Nelson et al. (2015), provided causal evidence that benevolent acts lead to increased positive affect and meaningfulness of the experience.

**Additional results for convergent and divergent validity**

***Psychological Need Satisfaction and Frustration Scale (PNSF)***

The PNSF instrument examines three basic psychological needs (autonomy, relatedness, competence), which have a corresponding factor within the WB-Pro (autonomy, positive relations, competence), specifically in relation to each basic need the instrument evaluates a satisfaction level and a frustration level. As expected, the WB-Pro factors were more strongly and positively related to the corresponding need satisfaction dimensions (.60, .78, .44) and less strongly and negatively related to the corresponding need frustration dimensions (-.41, -.56, -.32). Additional positive associations were found with non-corresponding (but somehow conceptually related) factors (e.g., relatedness with empathy =.49; competence satisfaction with engage =.72). Nonetheless, also unexpected results were found, for example the competence satisfaction scale was more associated with other conceptually related factors (e.g., clear thinking .67) than with the corresponding competence factor (.44), the autonomy satisfaction was more positively associated with the engage factor (.72) than the autonomy factor (.60). These results suggest that at least for the Italian versions of the instruments, the content of the two scales do not overlap completely, and the PNSF factors tend to be wider in content and less specific in comparison to the WB-Profile scales.

***Big Five Inventory (BFI)***

In relation to overall measures of well-being, it has been noted that correlations with the Big Five factors should be only modest in size. For example, in relation to an overall measure of eudaimonic well-being, Waterman and colleagues (2010) noted that “those high on extraversion may be more likely to pursue potentials involving social activities whereas those low on this personality factor would be more likely to develop potentials in other domains. However, whether or not the person chooses to pursue eudaimonic potentials in some domain should be largely independent of their standing on any particular trait” (p. 47). Things are slightly different for a multidimensional instrument such as the WB-Pro under examination here, therefore it is expected that some of the 15 WB-pro factors might be more associated with some dimensions of personality (e.g., positive relations with extraversion or agreeableness) but not others (positive relations with conscientiousness). Marsh et al. (2020), using the NEO-Five-Factor Inventory (Costa & McCrea, 1992b; Marsh et al., 2010), found that openness was most strongly associated with prosocial behavior and engagement, conscientiousness resulted most highly related to competence and clear thinking; extraversion was most highly associated with positive emotions and engagement; agreeableness was most highly correlated with prosocial behavior and empathy; and neuroticism was most highly correlated (negatively) with emotional stability, as well as with resilience, positive emotions, and self-acceptance.

As it was in Marsh et al.’s study, most Big Five factors showed from modest to moderate correlations with the WB-Pro factors confirming the discriminant validity of WB-Pro with BFI. Consciousness and Extraversion resulted the factors most associated with the WB-Pro factors (mean correlation for both factors .44, s.d. = 0.03), with the highest association with clear thinking for Consciousness (.62) and with vitality (.81) for Extraversion, which can be considered a component of Extraversion from a theoretical point of view. Neuroticism was moderately and negatively correlated with WB-Pro factors (mean correlation .41, s.d. = 0.04), with a high negative association with the emotional stability scale of the WB-Pro (-.86), which was theoretically expected. Openness showed moderate correlations with WB-Pro (mean correlation .32, s.d. = 0.04), with the highest associations with engage (.41). Agreeableness resulted moderately associated to WB-Pro factors (mean correlation .30, s.d. = 0.03), with the highest associations with empathy (.65) and prosocial behavior (.70), which are theoretically connected constructs.

***Single items***

The WB-Pro factors showed significant and positive associations with Life satisfaction item, even though the correlations were mostly modest, with the highest association with Positive emotions (.47) and Optimism (.41). The associations with Happiness item were not all statistically significant and mostly weak in strength, with the highest correlations again with Positive emotions (.23) and Optimism (.23). The correlation with General health item were overall small and some were non-significant at all; the highest correlations were with Positive emotions (.21) and Vitality (.19). No substantial association was found between sleep items or physical activity item and WB-Pro factors, with the only exception of a modest association between physical activity and Vitality. Therefore, overall, the patters of associations were close to those found by Marsh et al.’s study (2020) but the strength of the correlations was weaker.

***Background variables***

As it has been done in Marsh et al.’ study (2020), a set of background variables was regressed on the 15 WB-Pro factors. For many of the background variables, particularly gender and age, we found some positive and other negative relations with the WB-Pro factors, thus supporting the importance of having multidimensional instruments, since with univariate instruments these differences would have been levelled off. As in Marsh et al.’s 2020, males reported higher scores in Emotional stability, Self-acceptance, Vitality, and females reported higher scores in Empathy. In our sample males had higher scores on Optimism, and females on Positive emotions and Meaning, whereas no difference was found on the remaining scales. Several WB-Pro factors increased with age (Empathy, Meaning, Self-acceptance, Resilience and to a lesser extent Emotional stability and Clear thinking), other factors decreased with age (Prosocial behavior, Positive emotions, Optimism but also Autonomy). To better understand age effects, we also examined quadratic effects. An inverted U-shape effect was found in relation to autonomy, competence, optimism, positive relations and prosocial behaviour, with an initial increment in young adults, then a plateau and finally a decrement in older age. Positive quadratic effects (U-shape) were found for emotional stability, empathy, meaning, resilience, and self-acceptance, with an initial decrement in young adults, then a plateau and finally an increment in older age. In relation to education, individuals with higher educational levels showed higher scores on Emotional stability, Meaning and Vitality, and lower scores on Optimism and Autonomy. Also being married resulted positively associated with some WB-Pro scales (e.g. with Empathy r = .285), but negatively associated with other scales (e.g. with Autonomy = -.205). Finally, also some interaction effects were found, for example the gender differences favouring males in Self-acceptance and females in Meaning tend to decline with age, also it seems that youngster females experience more Positive emotions, whereas in older age are males to experience more Positive emotions.

***Links with other measures of well-being***

To better examine the interrelation between the WB-Pro and the other two instruments used in this study to evaluate well-being, following Marsh et al.’s (2020) procedure we examined the relations between Individual items from the WEMWBS and The Flourishing, and the 15 WB-Pro Factors. Based on the a-priori classification described by Marsh and colleagues, in which each one of the 8 Flourishing items and each one of the 14 WEMWBS were associated to one or more of the WB-Pro factors, we contrasted the unidimensional models of WEMWBS and The Flourishing and the overall multidimensional model in which the items of WEMWBS and Flourishing scales were absorbed into WB-Pro factors. It should be noted that the Italian version of the WEMWBS does not include items 4 and 12; nonetheless, to allow comparability with the original scale we maintained the original numbering of the items, this is why items 4 and 12 cannot be found in Table S3 (Supplemental materials). Fit indices of the model including the WB-Pro plus a general Flourishing factor and a general WEMWBS (χ^2^ = 6827.92, d.f. = 1609, scf = 1.26, CFI = .901, TLI = .859, RMSEA = .047) factor were poorer than those of a model in which items of the WEMWBS and the Flourishing were absorbed in the 15 WB-Pro factors (χ^2^ = 3556.49, d.f. = 1361, scf = 1.21, CFI = .958, TLI = .930, RMSEA = .033), thus supporting the latter model. Moreover, the majority of the WEMWBS and Flourishing items properly loaded on some of the WB-Pro factors, confirming that even though WEMWBS and Flourishing are often used as unidimensional scales, they can be considered indeed multidimensional in nature (see Table S3). Also, as already noted by Marsh and colleagues (2020, it is interesting that WEMWBS and Flourishing items do not cover exactly the same dimensions of well-being. For example, in our study, Competence is an area covered by the Flourishing scale but not the WEMWBS, whereas Empathy is covered by the WEMWBS and not by the Flourishing scale. Also, some dimensions measured by the WB-Pro are not properly covered by WEMWBS and Flourishing (e.g., Self-acceptance).

Table S1. Factor Loadings for the ESEM model of the WB-Profile

| item | ***autonomy*** | ***think clear*** | ***competence*** | ***emotional stability*** | ***empathy*** | ***engage*** | ***meaning*** | ***optimism*** | ***positive emotions*** | ***positive relations*** | ***prosocial behavior*** | ***resilience*** | ***self-acceptance*** | ***self-esteem*** | ***vitality*** |
| --- | --- | --- | --- | --- | --- | --- | --- | --- | --- | --- | --- | --- | --- | --- | --- |
| WB7 | **.74** | .03 | -.01 | -.03 | .01 | .02 | -.02 | -.02 | .02 | -.01 | -.04 | .11 | .03 | .05 | .03 |
| WB12 | **.89** | .06 | .00 | .01 | -.01 | .02 | .00 | .07 | -.03 | .06 | .02 | -.06 | .00 | -.03 | .01 |
| WB23 | **.70** | .04 | -.04 | -.01 | -.01 | .08 | .00 | -.03 | .08 | .03 | .04 | -.03 | .09 | .01 | -.02 |
| WB29 | .11 | **.15** | .17 | .11 | -.09 | .11 | .08 | -.09 | -.03 | .12 | .09 | .03 | .18 | .07 | .03 |
| WB46 | .01 | **.86** | .12 | .05 | .03 | -.05 | -.06 | .06 | .07 | .02 | -.03 | .00 | -.03 | .02 | .00 |
| WB36 | .01 | **.73** | .12 | -.01 | .06 | .07 | .04 | -.04 | .03 | .00 | .00 | .01 | .05 | -.07 | .03 |
| WB5 | -.01 | .22 | **.45** | -.08 | -.05 | .04 | .07 | .09 | -.13 | .07 | .05 | .03 | .08 | .23 | .05 |
| WB18 | .00 | .10 | **.34** | .06 | -.06 | .39 | .00 | .00 | -.04 | -.05 | .07 | .01 | .01 | .29 | .05 |
| WB17 | .02 | .13 | **.37** | .04 | -.01 | .28 | .01 | .02 | -.01 | -.05 | .01 | .03 | -.01 | .35 | .04 |
| WB44 | -.05 | .01 | -.06 | **.88** | .09 | .02 | -.03 | .00 | .03 | -.02 | -.09 | -.07 | .01 | .01 | .00 |
| WB10 | -.01 | .00 | .06 | **.74** | .05 | .07 | -.06 | .02 | -.02 | .05 | .00 | -.02 | .04 | -.08 | -.02 |
| WB39 | .00 | .10 | -.04 | **.50** | -.15 | -.12 | .18 | -.07 | .04 | .02 | .06 | .12 | .14 | .09 | .03 |
| WB43 | -.03 | -.01 | -.10 | .10 | **.54** | .01 | .01 | -.04 | -.16 | -.11 | .03 | .02 | .04 | .12 | -.02 |
| WB41 | -.01 | .00 | -.02 | .04 | **.49** | -.05 | -.03 | -.01 | -.03 | .02 | .26 | .01 | -.01 | .17 | -.07 |
| WB4 | -.04 | -.04 | -.06 | -.12 | **.75** | .01 | .09 | -.04 | -.01 | .00 | -.06 | -.02 | -.03 | -.05 | .00 |
| WB24 | .01 | -.05 | -.09 | -.07 | **.84** | .03 | -.07 | -.03 | .02 | -.01 | -.13 | -.01 | -.01 | -.05 | .01 |
| WB35 | .03 | .09 | .20 | .01 | .02 | **.73** | .12 | -.01 | .06 | -.02 | .04 | .05 | -.02 | -.20 | -.03 |
| WB13 | .12 | .10 | .24 | .05 | .03 | **.48** | .14 | -.03 | -.03 | .03 | -.03 | .03 | .01 | -.20 | .12 |
| WB19 | -.02 | -.07 | .24 | -.04 | .05 | **.65** | -.01 | .08 | .14 | .03 | -.02 | .01 | -.01 | .12 | .00 |
| WB2 | -.08 | -.06 | .23 | .06 | .00 | -.01 | **.69** | .02 | .11 | .13 | -.01 | .02 | -.06 | -.06 | -.04 |
| WB33 | .01 | .01 | -.02 | -.01 | .03 | .24 | **.57** | .11 | -.01 | -.01 | -.01 | -.02 | .13 | .00 | -.03 |
| WB38 | .04 | .08 | -.23 | .06 | .08 | .00 | **.90** | .03 | -.06 | -.01 | .02 | .04 | -.16 | .14 | .05 |
| WB45 | .01 | .03 | -.08 | -.04 | .03 | .07 | .05 | **.73** | .06 | .00 | .02 | -.01 | .08 | .06 | -.01 |
| WB3 | -.01 | -.03 | .15 | -.06 | -.06 | -.06 | .14 | **.80** | .05 | .05 | -.01 | -.04 | .05 | -.01 | .01 |
| WB11 | .02 | -.08 | -.02 | .09 | .01 | .02 | -.03 | **.79** | -.03 | -.03 | .01 | .07 | .09 | -.03 | .07 |
| WB27 | -.01 | -.03 | -.10 | .04 | -.04 | .05 | .03 | -.02 | **.65** | .11 | .05 | .07 | -.06 | .05 | .19 |
| WB48 | .04 | .07 | -.09 | .05 | -.06 | .01 | .00 | .05 | **.85** | -.05 | .06 | .04 | -.06 | .07 | .00 |
| WB42 | .05 | .01 | -.07 | -.02 | .03 | .09 | .02 | .06 | **.73** | .04 | .00 | .02 | .02 | .10 | -.07 |
| WB28 | .00 | .02 | .01 | .00 | -.04 | -.04 | .06 | -.07 | .02 | **.69** | .01 | -.03 | .06 | .11 | -.01 |
| WB26 | -.06 | .03 | -.14 | -.04 | -.01 | .10 | -.09 | .03 | -.05 | **.98** | -.05 | .06 | .02 | .02 | .00 |
| WB1 | -.02 | .01 | .08 | .03 | -.01 | -.03 | .03 | -.03 | .03 | **.63** | .03 | -.02 | -.06 | -.11 | -.03 |
| WB20 | .11 | .04 | -.09 | .06 | .03 | -.06 | .07 | .05 | .03 | **.56** | -.04 | -.09 | .03 | .00 | .02 |
| WB37 | -.02 | .06 | .01 | -.02 | .07 | -.03 | .05 | .06 | -.01 | -.01 | **.79** | .00 | .01 | -.03 | .03 |
| WB40 | .05 | .00 | .00 | .07 | .09 | -.04 | -.01 | -.06 | .09 | .02 | **.74** | -.03 | .00 | -.01 | -.02 |
| WB31 | -.01 | -.01 | .08 | -.07 | .09 | .04 | -.03 | .02 | .03 | -.01 | **.75** | -.01 | .08 | -.08 | .02 |
| WB6 | -.02 | .02 | .01 | -.02 | .04 | -.02 | .00 | .01 | .03 | .02 | -.04 | **.85** | .06 | .02 | .00 |
| WB34 | -.02 | .03 | -.04 | .09 | -.01 | .09 | -.03 | .07 | .09 | -.02 | .01 | **.66** | .14 | -.07 | -.04 |
| WB9 | .05 | -.02 | .06 | .00 | .02 | .00 | .06 | -.05 | .02 | -.04 | .00 | **.81** | .05 | .00 | .05 |
| WB22 | -.03 | .07 | -.06 | -.04 | .06 | -.05 | -.02 | -.04 | -.02 | -.02 | -.05 | .01 | **.87** | .01 | -.02 |
| WB21 | -.01 | .00 | .07 | .01 | .07 | .01 | -.14 | .04 | -.12 | .17 | .12 | .08 | **.34** | .07 | -.01 |
| WB14 | .12 | .03 | .05 | .18 | -.01 | -.05 | -.05 | .21 | -.07 | -.04 | .01 | .19 | **.30** | .02 | .02 |
| WB32 | -.01 | .04 | -.07 | .02 | -.06 | .00 | .07 | -.03 | .06 | -.05 | -.01 | -.09 | **.87** | .00 | .02 |
| WB15 | .06 | -.10 | .33 | .00 | .05 | -.07 | .12 | -.01 | .07 | .01 | -.02 | -.03 | .10 | **.59** | .06 |
| WB16 | .06 | -.03 | .28 | .03 | .21 | -.14 | -.03 | .03 | .13 | .02 | -.09 | .02 | .04 | **.71** | .03 |
| WB47 | -.05 | .18 | .26 | .06 | .01 | -.01 | .04 | .05 | .07 | .06 | .04 | .01 | .07 | **.46** | -.03 |
| WB30 | -.03 | .03 | -.04 | .00 | -.03 | .06 | .01 | .02 | .09 | .02 | .03 | .00 | .04 | .02 | **.79** |
| WB25 | -.04 | .04 | .01 | .00 | .03 | .03 | -.03 | .07 | .02 | -.01 | .02 | -.02 | .05 | -.04 | **.86** |
| WB8 | .10 | -.02 | .12 | .04 | .01 | -.01 | .00 | -.01 | .02 | .01 | -.01 | .04 | -.02 | .04 | **.75** |

Table S2. Latent factor correlations among the 15 WB-Profile factors

|  | ***autonomy*** | ***clear thinking*** | ***competence*** | ***emotional stability*** | ***empathy*** | ***engage*** | ***meaning*** | ***optimism*** | ***positive emotions*** | ***positive relations*** | ***prosocial behavior*** | ***resiliency*** | ***self-acceptance*** | ***self-esteem*** | ***vitality*** |
| --- | --- | --- | --- | --- | --- | --- | --- | --- | --- | --- | --- | --- | --- | --- | --- |
| ***autonomy*** | 1 | .386 | .287 | .366 | .238 | .484 | .449 | .484 | .548 | .352 | .253 | .466 | .565 | .290 | .518 |
| ***think clear*** |  | 1 | .184 | .443 | .321 | .697 | .513 | .482 | .454 | .298 | .378 | .433 | .548 | .470 | .446 |
| ***competence*** |  |  | 1 | .192 | .309 | .134 | .404 | .282 | .420 | .357 | .182 | .233 | .290 | .018 | .265 |
| ***emotional stability*** | |  |  | 1 | .218 | .468 | .363 | .557 | .487 | .193 | .209 | .546 | .549 | .359 | .414 |
| ***empathy*** |  |  |  |  | 1 | .360 | .404 | .312 | .385 | .451 | .597 | .184 | .373 | .197 | .302 |
| ***engage*** |  |  |  |  |  | 1 | .582 | .586 | .559 | .354 | .406 | .462 | .600 | .674 | .563 |
| ***meaning*** |  |  |  |  |  |  | 1 | .680 | .659 | .484 | .329 | .407 | .574 | .446 | .508 |
| ***optimism*** |  |  |  |  |  |  |  | 1 | .701 | .359 | .211 | .539 | .521 | .397 | .586 |
| ***positive emotions*** | |  |  |  |  |  |  |  | 1 | .558 | .301 | .525 | .647 | .407 | .701 |
| ***positive relations*** | |  |  |  |  |  |  |  |  | 1 | .445 | .247 | .438 | .261 | .335 |
| ***prosocial behavior*** | |  |  |  |  |  |  |  |  |  | 1 | .134 | .360 | .324 | .235 |
| ***resiliency*** |  |  |  |  |  |  |  |  |  |  |  | 1 | .543 | .345 | .552 |
| ***self-acceptance*** | |  |  |  |  |  |  |  |  |  |  |  | 1 | .498 | .540 |
| ***self-esteem*** |  |  |  |  |  |  |  |  |  |  |  |  |  | 1 | .350 |
| ***vitality*** |  |  |  |  |  |  |  |  |  |  |  |  |  |  | 1 |

Table S3. Effects of predictor background variables on the 15 WB factors and on the 5- and 15-item short versions of the WB-Profile

|  | ageL | ageQ | male | education | married | male x age | male x education | education x age | male x married | married x age |
| --- | --- | --- | --- | --- | --- | --- | --- | --- | --- | --- |
| Autonomy | -.212 | -.117 | .004 | -.106 | -.206 | .025 | .045 | -.019 | .010 | -.213 |
| Clear thinking | .112 | .082 | -.036 | .090 | .128 | .014 | .038 | .009 | .008 | .060 |
| Competence | -.074 | -.083 | -.046 | .036 | -.072 | .013 | -.027 | .004 | .045 | -.034 |
| Emotional stability | .135 | .105 | .173 | .162 | .010 | -.059 | -.070 | .033 | .007 | .050 |
| Empathy | .342 | .282 | -.249 | .059 | .285 | -.028 | -.065 | .094 | .024 | .159 |
| Engagment | -.037 | -.014 | .016 | -.104 | -.102 | .003 | -.019 | -.113 | -.034 | -.029 |
| Meaning | .207 | .221 | -.136 | .119 | .106 | -.099 | -.039 | .013 | -.024 | .105 |
| Optimism | -.186 | -.192 | .211 | -.130 | .045 | .032 | .051 | -.038 | -.083 | .010 |
| Psotive motions | -.114 | -.041 | -.197 | -.059 | .003 | .163 | .098 | .016 | .073 | -.077 |
| Positive realtions | -.184 | -.199 | -.021 | .033 | -.099 | .008 | .033 | -.063 | .010 | -.072 |
| Prosocial behavior | -.233 | -.140 | -.067 | -.081 | -.224 | .040 | .056 | -.014 | -.007 | -.101 |
| Resilience | .190 | .129 | .001 | .027 | .152 | -.017 | -.028 | .015 | -.020 | .089 |
| Self-acceptance | .199 | .116 | .156 | -.004 | .143 | -.111 | -.035 | .067 | -.044 | .173 |
| Self-esteem | .008 | -.056 | .062 | .053 | -.037 | -.008 | -.002 | .021 | .016 | -.099 |
| Vitality | .005 | -.023 | .138 | .120 | -.039 | .017 | -.150 | .033 | .068 | .053 |
| WB-Pro 15 | .131 | .080 | .063 | .109 | .115 | -.001 | -.064 | .016 | .000 | .080 |
| WB-Pro 5 | .135 | .071 | .085 | .155 | .095 | -.014 | -.092 | .049 | .019 | .094 |

*^Note^*^. ageL = age linear; ageQ = age quadratic. Each of the WB-Pro15 scales entered in the structural equation model was represented by a latent factor and regressed on the ten predictor variables. All first-order predictor variables in the model were standardized and all interaction terms were the product of standardized predictor variables.^

Table S4. Items of WEMWBS and Flourishing scales absorbed into the 15 WB-Profile factors

|  | WE1 | WE2 | WE3 | WE5 | WE6 | WE7 | WE8 | WE9 | WE10 | WE11 | WE13 | WE14 | FL1 | FL2 | FL3 | FL4 | FL5 | FL6 | FL7 | FL8 |
| --- | --- | --- | --- | --- | --- | --- | --- | --- | --- | --- | --- | --- | --- | --- | --- | --- | --- | --- | --- | --- |
| ***Autonomy*** | -.026 | -.050 | .040 | -.054 | -.015 | .025 | .125 | .013 | .051 | .261 | .103 | .053 | -.059 | -.102 | -.030 | -.107 | -.069 | -.016 | -.046 | -.055 |
| ***Think Clear*** | .012 | .089 | .008 | -.009 | .174 | .289 | **.429** | .081 | .187 | **.411** | **.301** | -.009 | -.048 | -.096 | .012 | -.155 | .198 | -.029 | -.119 | -.013 |
| ***Competence*** | .097 | .128 | -.074 | -.116 | .084 | .137 | .071 | -.090 | .171 | .110 | .137 | -.088 | .080 | -.025 | .251 | .104 | **.659** | **.358** | .175 | .198 |
| ***Emotional Stability*** | -.062 | -.052 | .228 | -.035 | -.192 | .038 | .165 | -.070 | -.002 | -.024 | -.075 | .025 | .024 | -.004 | .013 | -.040 | -.100 | .039 | -.074 | .051 |
| ***Empathy*** | .101 | .155 | -.031 | **.372** | .081 | -.006 | .022 | **.420** | -.041 | -.063 | .123 | -.036 | .051 | .000 | .088 | .125 | -.181 | -.109 | -.052 | -.115 |
| ***Engage*** | .162 | .222 | .127 | .244 | **.394** | .231 | **.326** | .230 | .139 | .178 | .295 | .231 | **.372** | .244 | **.533** | .113 | .125 | .084 | -.049 | .005 |
| ***Meaning*** | .118 | .106 | -.105 | -.035 | -.060 | .029 | -.027 | -.103 | -.002 | -.030 | -.145 | -.090 | **.561** | .072 | **.333** | .104 | .140 | .158 | .283 | .101 |
| ***Optimism*** | **.672** | .142 | .118 | .033 | .113 | .042 | -.058 | .045 | .221 | .093 | .153 | .080 | .089 | -.060 | -.119 | -.010 | .022 | -.044 | **.764** | -.003 |
| ***Positive Emotions*** | .057 | .033 | **.365** | .050 | .208 | -.047 | -.080 | -.072 | -.024 | -.197 | -.024 | **.624** | .164 | .228 | .154 | .138 | -.021 | .146 | -.019 | -.039 |
| ***Positive Relations*** | .054 | .053 | .042 | .235 | -.075 | .037 | .076 | **.315** | -.036 | .045 | .029 | .114 | .113 | **.674** | .279 | .183 | .046 | .238 | .008 | **.329** |
| ***Prosocial Behavior*** | -.054 | -.034 | -.065 | .135 | .016 | .034 | -.032 | .277 | -.102 | .036 | .105 | -.001 | -.031 | .045 | .012 | **.484** | .178 | .153 | .030 | .075 |
| ***Resiliency*** | -.008 | .063 | .001 | .007 | .121 | **.355** | .109 | .017 | .056 | .036 | -.042 | .035 | -.035 | -.060 | -.025 | -.002 | .001 | -.021 | -.019 | .084 |
| ***Self-Acceptance*** | -.047 | -.019 | .027 | -.123 | -.062 | -.003 | -.056 | -.076 | .192 | -.094 | -.198 | -.177 | -.027 | .136 | .109 | .054 | .152 | .071 | .076 | .169 |
| ***Self-Esteem*** | .064 | .239 | .089 | .007 | .106 | .177 | .124 | -.025 | **.436** | .249 | .019 | .140 | .058 | .018 | -.017 | .110 | **.398** | **.343** | .058 | .282 |
| ***Vitality*** | -.062 | .032 | -.044 | -.032 | **.317** | -.080 | -.052 | -.074 | .031 | .041 | .181 | .041 | .015 | -.024 | .069 | .020 | -.003 | -.059 | -.002 | -.017 |

*^Note. Factor loadings higher than .30 are in bold^*

**Additional references**

Abbott, R.A, Ploubidis, G. B., Huppert F. A., Kuh, D., Wadsworth, M. E., & Croudace, T. J. (2006). Psychometric evaluation and predictive validity of Ryff's psychological wellbeing items in a UK birth cohort sample of women. *Health Qual Life Outcomes, 4(1)*,76.

Abdallah, T. (1998). The Satisfaction with Life Scale (SWLS): Psychometric properties in an Arabic-speaking sample. *International Journal of Adolescence and Youth*, *7*(2), 113-119.

Aglozo, E. Y., Akotia, C. S., Osei-Tutu, A., & Annor, F. (2021). Spirituality and subjective well-being among Ghanaian older adults: Optimism and meaning in life as mediators. *Aging & Mental Health*, *25*(2), 306–315. doi:10.1080/13607863.2019.1697203.

Aknin, L. B., Barrington-Leigh, C. P., Dunn, E. W., Helliwell, J. F., Burns, J., Biswas-Diener, R., Kemeza, I., Nyende, P., Ashton-James, C. E., & Norton, M. I. (2013). Prosocial spending and well-being: Cross-cultural evidence for a psychological universal. *Journal of Personality and Social Psychology, 104*(4), 635–652. [https://doi.org/10.1037/a0031578](https://psycnet.apa.org/doi/10.1037/a0031578)

Allik, J., Realo, A. (1997). Emotional experience and its relation to the five-factor model in Estonian. *Journal of Personality*, 65, 625-647.

Alloy, L. B., Abramson, L. Y., Whitehouse, W. G., Hogan, M. E., Panzarella, C., & Rose, D. T. (2006). Prospective incidence of first onsets and recurrences of depression in individuals at high and low cognitive risk for depression. *Journal of Abnormal Psychology, 115*(1), 145–156. [https://doi.org/10.1037/0021-843X.115.1.145](https://psycnet.apa.org/doi/10.1037/0021-843X.115.1.145)

Ahmed, I., Rehman, W., Ali, F., Ali, G., & Anwar, F. (2018). Predicting employee performance through organizational virtuousness: Mediation by affective well-being and work engagement. *Journal of Management Development*, *37*(6), 493–502. doi:10.1108/JMD-04-2017-0115.

Antaramian, S. (2015). Assessing psychological symptoms and well-being: Application of a dual-factor mental health model to understand college student performance. *Journal of Psychoeducational Assessment, 33*(5), 419-429. doi:10.1177/0734282914557727.

Bal, P. M. (2020). Why we should stop measuring performance and well-being. *Zeitschrift Für Arbeits- Und Organisationspsychologie, 64*(3), 196–200. doi:10.1026/0932-4089/a000333.

Bandura, A. (1997). *Self-efficacy: The exercise of control*. New York, NY: Freeman.

Balatsky, G., & Diener, E. (1993). Subjective well-being among Russian students. *Social Indicators Research*, *28*(3), 225-243.

Bajaj, B., & Pande, N. (2016). Mediating role of resilience in the impact of mindfulness on life satisfaction and affect as indices of subjective well-being. *Personality and Individual Differences, 93,* 63–67. [https://doi.org/10.1016/j.paid.2015.09.005](https://psycnet.apa.org/doi/10.1016/j.paid.2015.09.005)

Batson, C. D. (1991). *The Altruism Question Toward a Social Psychological Answer*. Hillsdale, NJ Erlbaum.

Bayani, A. A., Koocheky, A. M., & Goodarzi, H. (2007). The reliability and validity of the satisfaction with life scale. *Developmental Psychology*, *3*(11), 259-65.

Belzer, K.D., D’Zurilla, T.J., & Maydeu-Olivares, A. (2002). Social problem solving and trait anxiety as predictors of worry in a college student population. *Personality and Individual Differences*, *33*(4), 573-585. DOI: 10.1016/S0191-8869(01)00173-8

Ben-Zur, H. (2003). Happy adolescents: The link between subjective well-being, internal resources, and parental factors. *Journal of Youth and Adolescence, 32*(2), 67–79. [https://doi.org/10.1023/A:1021864432505](https://psycnet.apa.org/doi/10.1023/A:1021864432505)

Bhattarai, M., Jin, Y., Smedema, S. M., Cadel, K. R., & Baniya, M. (2021). The relationships among self‐efficacy, social support, resilience, and subjective well‐being in persons with spinal cord injuries. *Journal of Advanced Nursing, 77*(1), 221–230. doi:10.1111/jan.14573.

Bourgault, P., Lavoie, S., Paul-Savoie, E., Grégoire, M., Michaud, C., Gosselin, E., & Johnston, C. C. (2015). Relationship between empathy and well-being among emergency nurses. *Journal of Emergency Nursing*, *41*(4), 323-328.

Brajša-Žganec, A., Ivanović, D., & Lipovčan, L. K. (2011). Personality traits and social desirability as predictors of subjective well-being. *Psihologijske Teme*, *20*(2), 261–276.

Bryant, F. B., & Veroff, J. (2007). *Savoring: A new model of positive experience.* Lawrence Erlbaum Associates Publishers.

Burns, R.A., Anstey K.J., Windsor, T.D. (2011). Subjective well-being mediates the effects of resilience and mastery on depression and anxiety in a large community sample of young and middle-aged adults. *The Australian & New Zealand Journal of Psychiatry*, *45*(3), 240-248. doi: 10.3109/00048674.2010.529604

Burns, R. A., & Machin, M. A. (2009). Investigating the Structural Validity of Ryff’s Psychological Well-Being Scales Across Two Samples. *Social Indicators Research, 93,* 359-375.

Butler, J., & Kern, M. L. (2016). The PERMA-Profiler: A brief multidimensional measure of flourishing. *International Journal of Wellbeing, 6*(3).1-48. doi:10.5502/ijw.v6i3.526

Butkovic, A., Brkovic, I., & Bratko, D. (2002). Predicting Well-Being From Personality in Adolescents and Older Adults. *Journal of Happiness Studies, 13*(3), 455-467.

Cañero Pérez, M., Mónaco Gerónimo, E., & Montoya Castilla, I. (2019). Emotional intelligence and empathy as predictors of subjective well-being in university students. *European Journal of Investigation in Health, Psychology and Education*, *9*(1), 19-29.

Carmichael, C. L., Reis, H. T., & Duberstein, P. R. (2015). In your 20s it's quantity, in your 30s it's quality: the prognostic value of social activity across 30 years of adulthood. *Psychology and aging*, *30*(1), 95–105. https://doi.org/10.1037/pag0000014

Caron, J., & Liu, A. (2011). Factors Associated with Psychological Distress in the Canadian Population: A Comparison of Low-Income and Non Low-Income Sub-Groups. *Community Mental Health Journal*, *47*, 318-330. DOI: 10.1007/s10597-010-9306-4

Carrozzino, D., Christensen, K. S., Patierno, C., Woźniewicz, A., Møller, S. B., Arendt, I. M. T., ... & Cosci, F. (2022). Cross-cultural validity of the WHO-5 Well-being Index and Euthymia Scale: A clinimetric analysis. *Journal of Affective Disorders, 311*, 276-283.

Carvajal, D., Aboaja, A., & Alvarado, R. (2015). Validación de la Escala de bienestar mental de Wareick-Edinburgo, en Chile. *Revista de Salud Pública*, *19*(1), 13-21.

Carver, C. S., & Scheier, M. F. (2002). Optimism. In C. R. Snyder & S. Lopez (Eds.), Handbook of positive psychology (pp. 231–242). New York, NY: Oxford University Press.

Cenkseven-Ouml, F. (2013). Decision-making and problem-solving as a well-being indicator among adolescents. *Educational Research and Reviews*, *8*(11), 720-727.

Chamberlain, J. M., & Haaga, D. A. (2001). Unconditional self-acceptance and psychological health. *Journal of Rational-Emotive and Cognitive-Behavior Therapy*, *19*(3), 163-176.

Chandoevwit, W., Thampanishvong, K. (2016). Valuing Social Relationships and Improved Health Condition Among the Thai Population. *Journal of Happiness Studies, 17,*2167-2189 (2016). https://doi.org/10.1007/s10902-015-9690-0

Chang, E. C., D'Zurilla, T. J., & Sanna, L. J. (2009). Social Problem Solving as a Mediator of the Link Between Stress and Psychological Well-being in Middle-Adulthood. *Cognitive Therapy and Research, 33*, 33-49.

Chen, B., Vansteenkiste, M., Beyers, W., Boone, L., Deci, E. L., Van der Kaap-Deeder, J., . . . Verstuyf, J. (2015). Basic psychological need satisfaction, need frustration, and need strength across four cultures. *Motivation and Emotion*, *39*, 216–236. http://dx.doi.org/10.1007/s11031-014-9450-1

Cho, E., & Jeon, S. (2019). The role of empathy and psychological need satisfaction in pharmacy students’ burnout and well-being. *BMC Medical Education*, *19*(1), 1-12.

Choi, S., & Choi, S. (2016). Conceptualizing Ryff’s Psychological Well-being Model with Confucian Perspective. *Korean Journal of Sociology, 50,* 101-123.

Chopik, W. J. (2017). Associations among relational values, support, health, and well‐being across the adult lifespan. *Personal Relationships, 24*(2), 408–422. [https://doi.org/10.1111/pere.12187](https://psycnet.apa.org/doi/10.1111/pere.12187)

Clench-Aas, J., Nes, R. B., Dalgard, O., & Aarø, L. (2011). Dimensionality and measurement invariance in the Satisfaction with Life Scale in Norway. *Quality of Life Research, 20(8)*, 1307–1317. https://doi. org/ 10. 1007/ s11136- 011- 9859-x

Costea-Bărluțiu, C., Bălaș-Baconschi, C., & Hathazi, A. (2018). Romanian adaptation of the Ryff’s Psychological Well-Being Scale: Brief report of the factor structure and psychometric properties. *Journal of Evidence-Based Psychotherapies, 18*(1), 21-33. [https://doi.org/10.24193/jebp.2018.1.2](https://psycnet.apa.org/doi/10.24193/jebp.2018.1.2)

Crawford, J. R., & Henry, J. D. (2004). The Positive and Negative Affect Schedule (PANAS): Construct validity, measurement properties and normative data in a large non‐clinical sample. *British Journal of Clinical Psychology*, *43*(3), 245-265

Damásio, B. F., & Koller, S. H. (2015). How search for meaning interacts with complex categories of meaning in life and subjective well-being? *The Spanish Journal of Psychology*, *18*. doi:10.1017/sjp.2015.1.

Datu, J. A. D., & King, R. B. (2018). Subjective well-being is reciprocally associated with academic engagement: A two-wave longitudinal study. *Journal of School Psychology*, *69*, 100–110. doi:10.1016/j.jsp.2018.05.007.

Davis, M. H. (2006). Empathy. In *Handbook of the sociology of emotions* (pp. 443-466). Boston, MA: Springer.

Deci, E. L., & Ryan, R. M. (2000). The “what” and “why” of goal pursuits: Human needs and the self-determination of behavior. *Psychological Inquiry, 11*, 227–268. https://doi.org/10.1207/S15327965PLI1104_01

Deptula, D. P., Cohen, R., Phillipsen, L. C., & Ey, S. (2006) Expecting the best: The relation between peer optimism and social competence*. The Journal of Positive Psychology,* *1*(3), 130-141

Diener, E., Wirtz, D., Tov, W., Kim-Prieto, C., Choi, D., Oishi, S., & Biswas-Diener, R. (2010). New well-being measures: Short scales to assess flourishing and positive and negative feelings. *Social Indicators Research, 97,* 143-156. <http://dx.doi.org/10.1007/s11205-009-9493-y>

Di Fabio, A., & Gori, A. (2016). Measuring adolescent life satisfaction: psychometric properties of the satisfaction with life scale in a sample of Italian adolescents and young adults. *Journal of Psychoeducational Assessment*, *34*(5), 501-506.

Di Fabio, A., & Gori, A. (2020). Satisfaction with life scale among Italian workers: reliability, factor structure and validity through a big sample study. *Sustainability*, *12*(14), 5860.

Di Maggio, I., Ginevra, M. C., Nota, L., Soresi, S. (2016). Development and validation of an instrument to assess future orientation and resilience in adolescence. *Journal of Adolescence 51,* 114-122

Dovidio, J. F., Piliavin, J. A., Schroeder, D. A., & Penner, L. (2006). *The social psychology of prosocial behavior.* Mahwah, NJ: Lawrence Erlbaum Associates Publishers.

Duy, B., & Yıldız, M. A. (2019). The mediating role of self-esteem in the relationship between optimism and subjective well-being. *Current Psychology*, *38*(6), 1456–1463. doi:10.1007/s12144-017-9698-1.

Echeverría Errázuriz, G., Torres Sahli, M., Pedrals Gibbons, N., Padilla Pérez, O., Rigotti Rivera, A., & Britan Carreño, M. (2017). Validation of a Spanish version of the mental health continuum-short form questionnaire. *Psicothema, 29*(1), 96-102.

Eisenberg, D., Gollust, S. E., Golberstein, E., Hefner, J. L. (2007). Prevalence and correlates of depression, anxiety, and suicidality among university students. *The American Journal of Orthopsychiatry, 77*(4), 534-42. doi: 10.1037/0002-9432.77.4.534.

Fava, G. A., & Bech, P. (2016). The concept of euthymia. *Psychotherapy and Psychosomatics, 85*(1), 1-5.

Ferguson, S. J., & Goodwin, A. D. (2010). Optimism and well-being in older adults: The mediating role of social support and perceived control. *The International Journal of Aging & Human Development, 71*(1), 43–68. doi:10.2190/AG.71.1.c.

Fotiadis, A., Abdulrahman, K., & Spyridou, A. (2019). The mediating roles of psychological autonomy, competence and relatedness on work-life balance and well-being. *Frontiers in Psychology*, *10*, 1267-1273. doi:10.3389/fpsyg.2019.01267.

Fredrickson, B. L. (2001). The role of positive emotions in positive psychology: The broaden-and-build theory of positive emotions. *American Psychologist: Special Issue,* *56*, 218–226.

Freire, C., Ferradas, M. del M., Núñez, J. C., & Valle, A. (2017). Estructura factorial de las Escalas de Bienestar Psicológico de Ryff en estudiantes universitarios. *European Journal of Education and Psychology, 10(1)*, 1-8.

Galinha, I. C., Oishi, S., Pereira, C., Wirtz, D., & Esteves, F. (2013). The role of personality traits, attachment style, and satisfaction with relationships in the subjective well-being of Americans, Portuguese, and Mozambicans. *Journal of Cross-Cultural Psychology, 44*(3), 416–437. doi:10.1177/0022022112453317.

Gallardo-Peralta, L. P., Martinez, M. Á. M., & Del Moral, R. S. (2019). Validation of the Personal Wellbeing Index (PWI) for older Chilean adults. *International Psychogeriatrics*, *31*(11), 1679-1680.

Giangrasso, B. (2021). Psychometric properties of the PERMA-Profiler as hedonic and eudaimonic well-being measure in an Italian context. *Current Psychology*, *40*(3), 1175-1184.

Giuntoli, L., Ceccarini, F., Sica, C., & Caudek, C. (2017). Validation of the Italian Versions of the Flourishing Scale and of the Scale of Positive and Negative Experience. *Sage Open*, *7*(1), 2158244016682293.

Glaesmer, H., Grande, G., Braehler, E., & Roth, M. (2011). The German version of the satisfaction with life scale (SWLS). *European Journal of Psychological Assessment, 27*(2),127-132. [https://doi.org/10.1027/1015-5759/a000058.](https://doi.org/10.1027/1015-5759/a000058)

Goodarzi, A., Shokri, O., & Sharifi, M. (2015). Cognitive appraisals, coping strategies, optimism and subjective well-being. *Journal of Psychology, 18*(4), 346–364.

Gouveia, V. V., Milfont, T. L., Da Fonseca, P. N., & de Miranda Coelho, J. A. P. (2009). Life satisfaction in Brazil: Testing the psychometric properties of the satisfaction with life scale (SWLS) in five Brazilian samples. *Social Indicators Research*, *90*(2), 267-277.

Govindji, R., & Linley, P. A. (2007). Strengths use, self-concordance and well- being: Implications for strengths coaching and coaching psychologists. *International Coaching Psychology Review, 2*(2), 143-153.

Griffin, K. W., Botvin, G. J., Scheier, L. M., Epstein, J. A., & Doyle, M. M. (2002). Personal competence skills, distress, and well-being as determinants of substance use in a predominantly minority urban adolescent sample. *Prevention Science*, *3*(1), 23–33. doi:10.1023/A:1014667209130.

Grozdanovska, E. (2016). The relationship between national identity, subjective well-being and meaning in life. *Suvremena Psihologija*, *19*(1), 91–99. doi:10.21465/2016-sp-191-08.

Gutiérrez, M., & Tomás, J. M. (2019). The role of perceived autonomy support in predicting university students’ academic success mediated by academic self-efficacy and school engagement. *Educational Psychology*, *39*(6), 729–748. doi:10.1080/01443410.2019.1566519.

Helliwell, J. F., Layard, R., Sachs, J., & De Neve, J. E. (2020). *World happiness report 2020*.

He, F., Cao, R., Feng, Z., Guan, H., & Peng, J. (2013). The impacts of dispositional optimism and psychological resilience on the subjective well-being of burn patients: A structural equation modelling analysis. *PLoS ONE*, *8*(12).

Heffner, A. L., & Antaramian, S. P. (2016). The role of life satisfaction in predicting student engagement and achievement. *Journal of Happiness Studies: An Interdisciplinary Forum on Subjective Well-Being, 17*(4), 1681–1701. doi:10.1007/s10902-015-9665-1.

Heinitz, K., Lorenz, T., Schulze, D., & Schorlemmer, J. (2018). Positive organizational behavior: Longitudinal effects on subjective well-being. *PLoS ONE,* 13(6). doi:10.1371/journal.pone.0198588.

Heissel, A., Pietrek, A., Flunger, B., Fydrich, T., Rapp, M. A., Heinzel, S., & Vansteenkiste, M. (2019). The validation of the German basic psychological need satisfaction and frustration scale in the context of mental health. *European Journal of Health Psychology*. 25(4). https://doi.org/10.1027/2512-8442/a000017

Henn, C. M., Hill, C., & Jorgensen, L. I. (2016). An investigation into the factor structure of the Ryff Scales of Psychological Well-Being. *SA* *Journal of Industrial Psychology*, *42*(1), 1-12.

Hilleras, P. K., Jorm, A. F., Herlitz, A., & Winblad, B. (1998). Negative and positive affect among the very old: A survey on a sample age 90 years or older. *Research on Aging*, *20*(5), 593-610.

Hofer, J., & Busch, H. (2011). Satisfying one’s needs for competence and relatedness: Consequent domain-specific well-being depends on strength of implicit motives. *Personality and Social Psychology Bulletin*, *37*(9), 1147–1158. doi:10.1177/0146167211408329.

Hoffman, S., Rueda, H. A., & Lambert, M. C. (2019). Confirmatory factor analysis of the Warwick-Edinburgh Mental Wellbeing Scale among youth in Mexico. *International Social Work*, *62*(1), 309-315.

Holopainen, L., Lappalainen, K., Junttila, N., & Savolainen, H. (2012). The Role of Social Competence in the Psychological Well-being of Adolescents in Secondary Education. *Scandinavian Journal of Educational Research*, *56*(2), 199–212. doi:10.1080/00313831.2011.581683.

Hone, L., Jarden, A., & Schofield, G. (2014). Psychometric properties of the Flourishing Scale in a New Zealand sample. *Social Indicators Research*, *119*(2), 1031-1045.

Houben, M., Van Den Noortgate, W., & Kuppens, P. (2015). The relation between short-term emotion dynamics and psychological well-being: A meta-analysis. *Psychological Bulletin, 141*(4), 901–930. doi:10.1037/a0038822.

Hsu, H.-Y., Hsu, T.-L., Lee, K., & Wolff, L. (2017). Evaluating the Construct Validity of Ryff’s Scales of Psychological Well-Being Using Exploratory Structural Equation Modeling. *Journal of Psychoeducational Assessment, 35(6),* 633-638.

Hultell, D., & Gustavsson, J. P. (2008). A psychometric evaluation of the satisfaction with life scale in a Swedish nationwide sample of university students. *Personality and Individual Differences, 44*(5), 1070–1079. [https://doi.org/10.1016/j.paid.2007.10.030](https://psycnet.apa.org/doi/10.1016/j.paid.2007.10.030)

Huta, V., & Ryan, R. M. (2010). Pursuing pleasure or virtue: The differential and overlapping well-being benefits of hedonic and eudaimonic motives. *Journal of happiness studies, 11*(6), 735-762.

Joshanloo, M., Wissing, M. P., Khumalo, I. P., & Lamers, S. M. (2013). Measurement invariance of the Mental Health Continuum-Short Form (MHC-SF) across three cultural groups. Personality and Individual Differences, 55(7), 755-759.

Jovanović, V., Cummins, R. A., Weinberg, M., Kaliterna, L., & Prizmic-Larsen, Z. (2019). Personal wellbeing index: a cross-cultural measurement invariance study across four countries. *Journal of Happiness Studies*, *20*(3), 759-775.

Junça-Silva, A., Caetano, A., & Lopes, R. R. (2017). Daily uplifts, well-being and performance in organizational settings: The differential mediating roles of affect and work engagement. *Journal of Happiness Studies*, *18*(2), 591–606. doi:10.1007/s10902-016-9740-2.

Kachanoff, F. J., Taylor, D. M., Caouette, J., Khullar, T. H., & Wohl, M. J. A. (2019). The chains on all my people are the chains on me: Restrictions to collective autonomy undermine the personal autonomy and psychological well-being of group members. *Journal of Personality and Social Psychology, 116*(1), 141–165. [https://doi.org/10.1037/pspp0000177](https://psycnet.apa.org/doi/10.1037/pspp0000177)

Kállay, É. (2015). The investigation of the relationship between the meaning attributed to life and work, depression, and subjective and psychological well-being in Transylvanian Hungarian young adults. *Cognition, Brain, Behavior: An Interdisciplinary Journal*, *19*(1), 17–33.

Karaś, D., Cieciuch, J., & Keyes, C. L. (2014). The polish adaptation of the mental health continuum-short form (MHC-SF). Personality and Individual Differences, 69, 104-109.

Khan, A. (2013). Predictors of Positive Psychological Strengths and Subjective Well-Being Among North Indian Adolescents: Role of Mentoring and Educational Encouragement. *Social Indicators Research*, *114*(3), 1285–1293. doi:10.1007/s11205-012-0202-x.

Kobylińska, D., Zajenkowski, M., Lewczuk, K., Jankowski, K. S., & Marchlewska, M. (2020). The mediational role of emotion regulation in the relationship between personality and subjective well-being*. Current Psychology.* doi:10.1007/s12144-020-00861-7.

Konaszewski, K., Niesiobędzka, M. & Surzykiewicz, J. (2021). Factor structure and psychometric properties of a Polish adaptation of the Warwick–Edinburgh Mental Wellbeing Scale. *Health Qual Life Outcomes*, *19*, 70. <https://doi.org/10.1186/s12955-021-01716-w>

Krohne, H. W., Egloff, B., Kohlmann, C. W., & Tausch, A. (1996). Positive and negative affect schedule--German version. *Diagnostica*, *42*, 139-156.

Krok, D., & Telka, E. (2019). Optimism mediates the relationships between meaning in life and subjective and psychological well-being among late adolescents. *Current Issues in Personality Psychology*, *7*(1), 32–42. doi:10.5114/cipp.2018.79960.

Kuźma, B., Szulawski, M., Vansteenkiste, M., Cantarero, K. (2020). Polish Adaptation of the Basic Psychological Need Satisfaction and Frustration Scale. *Frontiers in Psychology*, 29, 10: 3034. doi: 10.3389/fpsyg.2019.03034.

Lamers, S. M., Westerhof, G. J., Bohlmeijer, E. T., ten Klooster, P. M., & Keyes, C. L. (2011). Evaluating the psychometric properties of the mental health continuum‐short form (MHC‐SF). Journal of clinical psychology, 67(1), 99-110.

Landa, R. J., Holman, K. C., O'Neill, A.H., & Stuart, E. A. (2011). Intervention targeting development of socially synchronous engagement in toddlers with autism spectrum disorder: a randomized controlled trial. *The Journal of Child Psychology and Psychiatry, 52*(1), 13-21. doi: 10.1111/j.1469-7610.2010.02288.x.

Lau, A. L., Cummins, R. A., & Mcpherson, W. (2005). An investigation into the cross-cultural equivalence of the Personal Wellbeing Index. *Social Indicators Research*, *72*(3), 403-430.

Lau, C., Chiesi, F., & Saklofske, D. H. (2019). The combinative role of traits cheerfulness and seriousness relating to resiliency and well-being: A moderated mediation model. *Personality and Individual Differences, 151,* 109515. doi:10.1016/j.paid.2019.109515.

Lemola, S., Räikkönen, K., Scheier, M. F., Matthews, K. A., Pesonen, A. K., Heinonen, K., Lahti, J., Komsi, N., Paavonen, J. E., & Kajantie, E. (2011). Sleep quantity, quality and optimism in children. *Journal of sleep research*, *20*(1), 12–20. https://doi.org/10.1111/j.1365-2869.2010.00856.x

Lewis, A. D., Huebner, E. S., Malone, P. S., & Valois, R. F. (2011). Life satisfaction and student engagement in adolescents. *Journal of Youth and Adolescence, 40*(3), 249–262. doi:10.1007/s10964-010-9517-6.

Li, Z., Yin, X., Jiang, S., Wang, M., & Cai, T. (2014). Psychological Mechanism of Subjective Well-Being: A Stable Trait or Situational Variability. *Social Indicators Research, 118*(2), 523–534. doi:10.1007/s11205-013-0449-x.

Liga, F., Ingoglia, S., Cuzzocrea, F., Inguglia, C., Costa, S., Coco, A. L., & Larcan, R. (2018). The basic psychological need satisfaction and frustration scale: Construct and predictive validity in the Italian context. *Journal of Personality Assessment*. *102*(1), 102-112. doi: 10.1080/00223891.2018.1504053

Lizano, E. L. (2021). Work engagement and its relationship with personal well-being: A cross-sectional exploratory study of human service workers. *Human Service Organizations: Management, Leadership & Governance*, *45*(4), 326–336. doi:10.1080/23303131.2021.1898071.

Lodi, E., Boerchi, D., Magnano, P., Patrizi, P. (2019). High-School Satisfaction Scale (H-Sat Scale): Evaluation of Contextual Satisfaction in Relation to High-School Students’ Life Satisfaction. *Behavioral Sciences*, *9*, 125-142.

Lopez, M.A., A. Gabilondo, M. Codony, C. Garcia-Forero, G. Vilagut, P. Castellvi, M. Ferrer and J. Alonso (2013). Adaptation into Spanish of the Warwick-Edinburgh Mental Well-Being Scale (WEMWBS) and preliminary validation in a student sample. *Quality of Life Research* *22*(5), 1099–104.

Lundman, B., Strandberg, G., Eisemann, M., Gustafson, Y., & Brulin, C. (2007). Psychometric properties of the Swedish version of the Resilience Scale. Scandinavian Journal of Caring Sciences, 21(2), 229-237. doi: 10.1111/j.1471-6712.2007.00461.x.

Luruli, K., Mostert, K., & Jacobs, M. (2020). Testing a structural model for study demands and resources, study engagement and well-being of first-year university students. *Journal of Psychology in Africa, 30*(3), 179–186. doi:10.1080/14330237.2020.1767925.

MacInnes, D. L. (2006). Self‐esteem and self‐acceptance: an examination into their relationship and their effect on psychological health. *Journal of Psychiatric and Mental Health Nursing*, *13*(5), 483-489.

Magnano, P., Lodi, E., Boerchi, D. (2020). The role of non-intellective competences and performance in college satisfaction. *Interchange, DOI:10.1007/s10780-019-09385-x*

Marsh, H. W., Huppert, F. A., Donald, J. N., Horwood, M. S., & Sahdra, B. K. (2020). The well-being profile (WB-Pro): Creating a theoretically based multidimensional measure of well-being to advance theory, research, policy, and practice. *Psychological Assessment, 32*(3), 294–313. [https://doi.org/10.1037/pas0000787](https://psycnet.apa.org/doi/10.1037/pas0000787)

Martela, F., & Ryan, R. M. (2016). Prosocial behavior increases well-being and vitality even without contact with the beneficiary: Causal and behavioral evidence. *Motivation and Emotion*, *40*(3), 351-357.

Masten, A. S., Tellegen, A. (2012). Resilience in developmental psychopathology: contributions of the Project Competence Longitudinal Study. *Development and Psychopathology, 24*(2), 345-61. doi: 10.1017/S095457941200003X.

Mavali, S., Mahmoodi, H., Sarbakhsh, P., & Shaghaghi, A. (2020). Psychometric properties of the Warwick–Edinburgh Mental Wellbeing Scale (WEMWBS) in the Iranian older adults. *Psychology Research and Behavior Management*, *13*, 693-700.

McIntyre, E., Saliba, A., & McKenzie, K. (2020). Subjective wellbeing in the Indian general population: a validation study of the Personal Wellbeing Index. *Quality of Life Research*, *29*(4), 1073-1081.

Meyer, B., Enström, M. K., Harstveit, M., Bowles, D. P., & Beevers, C. G. (2007). Happiness and despair on the catwalk: Need satisfaction, well-being, and personality adjustment among fashion models. *The Journal of Positive Psychology, 2*(1), 2–17. [https://doi.org/10.1080/17439760601076635](https://psycnet.apa.org/doi/10.1080/17439760601076635)

Morris, M., Burns, G., Periard, D., & Shoda, E. (2015). Extraversion-Emotional Stability Circumplex Traits and Subjective Well-Being. *Journal of Happiness Studies*, *16*(6), 1509–1523. doi:10.1007/s10902-014-9573-9.

Nalin, C. P., & de Freitas Pinho França, L. H. (2015). The importance of resilience for well-being in retirement. *Paidéia*, *25*(61), 191–199. doi:10.1590/1982-43272561201507.

Nartova-Bochaver, S., Donat, M., & Rüprich, C. (2019). Subjective well-being from a just-world perspective: A multi-dimensional approach in a student sample. *Frontiers in Psychology*, *10*. doi:10.3389/fpsyg.2019.01739.

Nelson, S. K., Della Porta, M. D., Bao, K. J., Lee, H. C., Choi, I., & Lyubomirsky, S. (2015). ‘It’s up to you’: Experimentally manipulated autonomy support for prosocial behavior improves well-being in two cultures over six weeks. *The Journal of Positive Psychology, 10*(5), 463–476. [https://doi.org/10.1080/17439760.2014.983959](https://psycnet.apa.org/doi/10.1080/17439760.2014.983959)

Nezu, A. M., D'Zurilla, T. J., Zwick, M. L., & Nezu, C. M. (2004). Problem-Solving Therapy for Adults. In E. C. Chang, T. J. D'Zurilla, & L. J. Sanna (Eds.), *Social problem solving: Theory, research, and training* (pp. 171–191). American Psychological Association. [https://doi.org/10.1037/10805-010](https://psycnet.apa.org/doi/10.1037/10805-010)

Ng, W. (2015). Processes underlying links to subjective well-being: Material concerns, autonomy, and personality. *Journal of Happiness Studies*, *16*(6), 1575–1591. doi:10.1007/s10902-014-9580-x.

Ng, W. (2017). Extending Traditional Psychological Disciplines to Positive Psychology: A View from Subjective Well-being. *Journal of Happiness Studies, 18*(5), 1553–1571. doi:10.1007/s10902-016-9782-5.

Ng, W., Russell Kua, W. S., & Kang, S.-H. (2019). The relative importance of personality, financial satisfaction, and autonomy for different subjective well-being facets. *The Journal of Psychology: Interdisciplinary and Applied*. *153*(7), 680–700. doi:10.1080/00223980.2019.1598928.

Nikolaev, B., Boudreaux, C. J., & Wood, M. (2020). Entrepreneurship and subjective well-being: The mediating role of psychological functioning. *Entrepreneurship Theory & Practice*, *44*(3), 557–586. doi:10.1177/1042258719830314.

Nišević, S., & Cigić, D. (2013). Ryff’s Psychological Well-Being Scales: Factor Structure of Serbian Version. *Current Topics in Neurology, Psychiatry & Borderline Disciplines/ Aktuelnosti iz Neurologije,* *Psihijatrije i Granicnih Podrucja, 21(3/4),* 8-17.

Nishimura, T., & Suzuki, T. (2016). Basic psychological need satisfaction and frustration in Japan: controlling for the big five personality traits. *Japanese Psychological Research*, *58*(4), 320-331.

O’Donnell, S. L., Chang, K. B., & Miller, K. S. (2013). Relations among autonomy, attribution style, and happiness in college students. *College Student Journal*, *47*(1), 228–234.

Olesen, M. H., Thomsen, D. K., & O’Toole, M. S. (2015). Subjective well-being: Above neuroticism and extraversion, autonomy motivation matters. *Personality and Individual Differences*, *77*, 45–49. doi:10.1016/j.paid.2014.12.033.

Ong, Z. X., Dowthwaite, L., Perez Vallejos, E., Rawsthorne, M., & Long, Y. (2021). Measuring Online Wellbeing: A Scoping Review of Subjective Wellbeing Measures. *Frontiers in psychology*, *12*, 616637. https://doi.org/10.3389/fpsyg.2021.616637

Orkibi, H., & Tuaf, H. (2017). School engagement mediates well-being differences in students attending specialized versus regular classes. *Journal of Educational Research, 110*(6), 675–682. doi:10.1080/00220671.2016.1175408.

Paradise, A. W., & Kernis, M. H. (2002). Self-esteem and psychological well-being: Implications of fragile self-esteem. *Journal of Social and Clinical Psychology*, *21*(4), 345-361.

Petrillo, G., Capone, V., Caso, D., & Keyes, C. L. (2015). The Mental Health Continuum–Short Form (MHC–SF) as a measure of well-being in the Italian context. Social indicators research, 121(1), 291-312.

Pezirkianidis, C., Stalikas, A., Lakioti, A., & Yotsidi, V. (2021). Validating a multidimensional measure of wellbeing in Greece: Translation, factor structure, and measurement invariance of the PERMA Profiler. *Current Psychology*, *40*(6), 3030-3047.

Piliavin, J. A. (2003). Doing well by doing good: Benefits for the benefactor. In C. L. M. Keyes & J. Haidt (Eds.), *Flourishing: Positive psychology and the life well-lived* (pp. 227–247). American Psychological Association. [https://doi.org/10.1037/10594-010](https://psycnet.apa.org/doi/10.1037/10594-010)

Poulin, V., Korner-Bitensky, N., Dawson, D. R., Bherer, L. (2012). Efficacy of executive function interventions after stroke: a systematic review. *Top Stroke Rehabilitation, 19*(2), 158-71. doi: 10.1310/tsr1902-158.

Puente-Díaz, R. & Cavazos, J. (2013). Personality factors, affect, and autonomy support as predictors of life satisfaction. *Universitas Psychologica, 12*(1), 41-53.

Rahkman Ardi, A. (2018). The Mental Health Continuum-Short Form: The structure and application for cross-cultural studies–a 38 nation study. The Journal of Clinical Psychology, 74(6), 1034-1052.

Rani, N. (2019). Analytical behavior approaches for employee using psychological capital and well-being. *IAHRW International Journal of Social Sciences Review*, *7*(4), 725–728.

Reivich, K., Gillham, J. E., Chaplin, T. M., & Seligman, M. E.P. (2013). From helplessness to optimism: The role of resilience in treating and preventing depression in youth. In S. Goldstein & R. B. Brooks (Eds.), *Handbook of resilience in children* (pp. 201–214). Springer Science + Business Media. [https://doi.org/10.1007/978-1-4614-3661-4_12](https://psycnet.apa.org/doi/10.1007/978-1-4614-3661-4_12)

Roberts, M. C., Brown, K. J., Johnson, R. J., & Reinke, J. (2002). Positive psychology for children: Development, prevention, and promotion. In C. R. Snyder & S. J. Lopez (Eds.), *Handbook of positive psychology* (pp. 663–675). New York, NY: Oxford University Press.

Robles, R., & Páez, F. (2003). Estudio sobre la traducción al español y las propiedades psicométricas de las escalas de afecto positivo y negativo (PANAS). *Salud Mental*, *26*(1), 69-75.

Rodriguez-Carvajal, R., Moreno-Jiménez, B., & van Dierendonck, D. (2010). Vitality and inner resources as relevant components of psychological well-being. *Psicothema*, *22*(1), 63-70.

Roepke, A. M., Jayawickreme, E., & Riffle, O. M. (2014). Meaning and health: A systematic review. *Applied Research in Quality of Life, 9*(4), 1055–1079. doi:10.1007/s11482-013-9288-9.

Ryan, J., Curtis, R., Olds, T., Edney, S., Vandelanotte, C., Plotnikoff, R., & Maher, C. (2019). Psychometric properties of the PERMA Profiler for measuring wellbeing in Australian adults. *PloS one*, *14*(12), e0225932.

Ryan, R. M., & Deci, E. L. (2001). On happiness and human potentials: A review of research on hedonic and eudaimonic well-being. Annual Review of Psychology, 52, 141-166. [https://doi.org/10.1146/annurev.psych.52.1.141](https://psycnet.apa.org/doi/10.1146/annurev.psych.52.1.141)

Ryan, R. M., & Deci, E. L. (2017). *Self-determination theory: Basic psychological needs in motivation, development, and wellness.* The Guilford Press. [https://doi.org/10.1521/978.14625/28806](https://psycnet.apa.org/doi/10.1521/978.14625/28806)

Ryan, R. M., Weinstein, N., Bernstein, J., Brown, K. W., Mistretta, L., Gagné, L. (2010). Vitalizing effects of being outdoors and in nature. *Journal of Environmental Psychology, 30*(2), 159-168

Ryan, R. M., & Frederick, C. M. (1997). On energy, personality and health: Subjective vitality as a dynamic reflection of well-being. *Journal of Personality, 65,*529-565.

Ryff, C. D. (2013). Eudaimonic well-being and health: Mapping consequences of self-realization. In A. S. Waterman (Ed.), *The best within us: Positive psychology perspectives on eudaimonia* (pp. 77–98). American Psychological Association.

[https://doi.org/10.1037/14092-005](https://psycnet.apa.org/doi/10.1037/14092-005)

Ryff, C. D., & Keyes, C. L. M. (1995). The structure of psychological well-being revisited. *Journal of personality and social psychology*, *69*(4), 719.

Sachs, J. (2003). Validation of the satisfaction with life scale in a sample of Hong Kong university students. *Psychologia*, *46*(4), 225-234.

Sandín, B., Chorot, P., Lostao, L., Joiner, T. E., Santed, M. A., & Valiente, R. M. (1999). Escalas PANAS de afecto positivo y negativo: validación factorial y convergencia transcultural. *Psicothema*, *11*(1), 37-51.

Satici, S. A. (2016). Psychological vulnerability, resilience, and subjective well-being: The mediating role of hope. *Personality and Individual Differences, 102*, 68–73.

Schulz, P., Schulte, J., Raube, S., Disouky, H., & Kandler, C. (2018). The Role of Leisure Interest and Engagement for Subjective Well-Being. *Journal of Happiness Studies, 19(*4), 1135–1150. doi:10.1007/s10902-017-9863-0.

Seligman, M. E., Steen, T. A., Park, N., & Peterson, C. (2005). Positive psychology progress: empirical validation of interventions. *American psychologist*, *60*(5), 410.

Seligman, M. E. P. (2011). *Flourish: A visionary new understanding of happiness and well-being.* New York, NY: Free Press.

Sheldon, K. M., Kasser, T., Houser-Marko, L., Jones, T., & Turban, D. (2005). Doing One’s Duty: Chronological Age, Felt Autonomy, and Subjective Well-Being. *European Journal of Personality*, *19*(2), 97–115. doi:10.1002/per.535.

Skevington, S. M. (1999). Measuring quality of life in Britain: introducing the WHOQOL-100. *Journal of Psychosomatic Research*, *47*(5), 449-459.

Silva, A.J., & Caetano, A. (2013). Validation of the flourishing scale and scale of positive and negative experience in Portugal. *Social Indicators Research*, *110*(2), 469-478.

Singh, K., Junnarkar, M., & Jaswal, S. (2016). Validating the flourishing scale and the scale of positive and negative experience in India. *Mental Health, Religion & Culture*, *19*(8), 943-954.

Sirigatti, S., Penzo, I., Iani, L., Mazzeschi, A., Hatalskaja, H., Giannetti, E., & Stefanile, C. (2013). Measurement invariance of Ryff’s psychological well-being scales across Italian and Belarusian students. *Social Indicators Research*, *113*(1), 67-80.

Siu, A. M. H., & Shek, D. T. L. (2010). Social problem solving as a predictor of well-being in adolescents and young adults. *Social Indicators Research, 95*(3), 393–406. [https://doi.org/10.1007/s11205-009-9527-5](https://psycnet.apa.org/doi/10.1007/s11205-009-9527-5)

Stănculescu, E. (2016). Structural equation modeling of the relation between self-esteem, optimism, presence of a sense of meaning in life, and subjective well-being. *Revista de Psihologie*, *62*(3), 227–240.

Suar, D., Jha, A. K., Das, S. S., & Alat, P. (2019). The structure and predictors of subjective well-being among millennials in India. *Cogent Psychology, 6*(1). doi:10.1080/23311908.2019.1584083.

Sumi, K. (2014). Reliability and validity of Japanese versions of the Flourishing Scale and the Scale of Positive and Negative Experience. *Social Indicators Research*, *118*(2), 601-615.

Swami, V., & Chamorro-Premuzic, T. (2009). Psychometric evaluation of the Malay satisfaction with life scale. *Social Indicators Research*, *92*(1), 25-33.

Tang, X., Duan, W., Wang, Z., & Liu, T. (2016). Psychometric evaluation of the simplified Chinese version of flourishing scale. *Research on Social Work Practice*, *26*(5), 591-599

Tay, L., Tan, K., Diener, E., Gonzalez, E. (2012). Social relations, health behaviors, and health outcomes: a survey and synthesis. *Applied Psychology: Health and Well-Being, 5*(1), 28-78. doi: 10.1111/aphw.12000.

Thøgersen-Ntoumani, C., Ntoumanis, N. A. (2007). Self-determination Theory approach to the study of body image concerns, self-presentation and self-perceptions in a sample of aerobic instructors. *Journal of Health Psychology, 12(*2), 301-15. doi: 10.1177/1359105307074267.

Tugade, M. M., & Fredrickson, B. L. (2004). Resilient Individuals Use Positive Emotions to Bounce Back From Negative Emotional Experiences. *Journal of Personality and Social Psychology, 86*(2), 320–333. [https://doi.org/10.1037/0022-3514.86.2.320](https://psycnet.apa.org/doi/10.1037/0022-3514.86.2.320)

Vázquez, C., Duque, A., & Hervás, G. (2013). Satisfaction with life scale in a representative sample of Spanish adults: validation and normative data. *The Spanish Journal of Psychology, 16*, E82. doi: 10.1017/sjp.2013.82. PMID: 24230945.

Vella-Brodrick, D., Park, N., & Peterson, C. (2009). Three Ways to Be Happy: Pleasure, Engagement, and Meaning—Findings from Australian and US Samples. *Social Indicators Research*, *90*(2), 165–179. doi:10.1007/s11205-008-9251-6.

Vera-Villarroel, P., Urzúa, A., Celis-Atenas, P. P. K., & Silva, J. (2012). Evaluation of subjective well-being: Analysis of the satisfaction with life scale in Chilean population. *Universitas Psychologica*, *11*(3), 719-727.

Villieux, A., Sovet, L., Jung, S. C., & Guilbert, L. (2016). Psychological flourishing: Validation of the French version of the Flourishing Scale and exploration of its relationships with personality traits. *Personality and Individual Differences*, *88*, 1-5

Wammerl, M., Jaunig, J., Mairunteregger, T., & Streit, P. (2019). The German version of the PERMA-Profiler: evidence for construct and convergent validity of the PERMA theory of well-being in German speaking countries. *Journal of Well-Being Assessment*, *3*(2), 75-96.

Watanabe, K., Kawakami, N., Shiotani, T., Adachi, H., Matsumoto, K., Imamura, K., ... & Kern, M. L. (2018). The Japanese Workplace PERMA-Profiler: a validation study among Japanese workers. *Journal of Occupational Health*, *60*(5), 383–393.

Wei, M., Shaffer, P., Young, S., & Zakalik, R. (2005). Adult attachment, shame, depression, and loneliness: The mediation role of basic psychological needs satisfaction. *Journal of Counseling Psychology*, *54*(4), 591–601.

Weinstein, N., & Ryan, R. M. (2010). When helping helps: Autonomous motivation for prosocial behavior and its influence on well-being for the helper and recipient. *Journal of Personality and Social Psychology, 98*(2), 222–244. [https://doi.org/10.1037/a0016984](https://psycnet.apa.org/doi/10.1037/a0016984)

Wicaksono, D. A., Roebianto, A., & Sumintono, B. (2018). Internal Validation of the Warwick Edinburgh Mental Wellbeing Scale: Rasch Analysis in the Indonesian Context. *Scandinavian Journal of Public Health*, *46*(7), 718-725. doi: 10.1177/1403494817735391.

Williamson, G. M., & Clark, M. S. (1989). Providing help and desired relationship type as determinants of changes in moods and self-evaluations. *Journal of Personality and Social Psychology, 56*(5), 722–734. [https://doi.org/10.1037/0022-3514.56.5.722](https://psycnet.apa.org/doi/10.1037/0022-3514.56.5.722)

WHO (1996). *WHOQOL-BREF Introduction, Administration, Scoring, and Generic Version of the Assessment*. World Health Organisation. Available online at <https://www.who.int/mental_health/media/en/76.pdf>

Yan, X., Su, J., Wen, Z., & Luo, Z. (2019). The role of work engagement on the relationship between personality and job satisfaction in Chinese nurses. *Current Psychology*, *38*(3), 873–878. doi:10.1007/s12144-017-9667-8.

Yu, S., Levesque-Bristol, C., & Maeda, Y. (2018). General need for autonomy and subjective well-being: A meta-analysis of studies in the US and East Asia. *Journal of Happiness Studies: An Interdisciplinary Forum on Subjective Well-Being, 19*(6), 1863–1882. [https://doi.org/10.1007/s10902-017-9898-2](https://psycnet.apa.org/doi/10.1007/s10902-017-9898-2)

Zambianchi, M. (2015). *La psicologia positiva*. Roma: Carocci.

Żemojtel‐Piotrowska, M., Piotrowski, J. P., Osin, E. N., Cieciuch, J., Adams, B. G., Ardi, R., ... & Maltby, J. (2018). The mental health continuum‐short form: The structure and application for cross‐cultural studies–A 38 nation study. Journal of Clinical Psychology, 74(6), 1034-1052.

Zubair, A., Kamal, A., & Artemeva, V. (2018). Mindfulness and resilience as predictors of subjective well-being among university students: A cross cultural perspective. *Journal of Behavioural Sciences*, *28*(2), 1–19.
